# Three-Dimensional Printing/Bioprinting and Cellular Therapies for Regenerative Medicine: Current Advances

**DOI:** 10.3390/jfb16010028

**Published:** 2025-01-16

**Authors:** Ana Catarina Sousa, Rui Alvites, Bruna Lopes, Patrícia Sousa, Alícia Moreira, André Coelho, José Domingos Santos, Luís Atayde, Nuno Alves, Ana Colette Maurício

**Affiliations:** 1 Departamento de Clínicas Veterinárias, Instituto de Ciências Biomédicas de Abel Salazar (ICBAS), Universidade do Porto (UP), Rua de Jorge Viterbo Ferreira, nº 228, 4050-313 Porto, Portugal; anacatarinasoaressousa@hotmail.com (A.C.S.); ruialvites@hotmail.com (R.A.); brunisabel95@gmail.com (B.L.); pfrfs_10@hotmail.com (P.S.); alicia.moreira.1998@gmail.com (A.M.); andrefmc17@gmail.com (A.C.); ataydelm@gmail.com (L.A.); 2 Centro de Estudos de Ciência Animal (CECA), Instituto de Ciências, Tecnologias e Agroambiente da Universidade do Porto (ICETA), Rua D. Manuel II, Apartado 55142, 4051-401 Porto, Portugal; 3 Associate Laboratory for Animal and Veterinary Science (AL4AnimalS), Av. Universidade Técnica, 1300-477 Lisboa, Portugal; 4 Instituto Universitário de Ciências da Saúde (CESPU), Instituto Universitário de Ciências da Saúde (IUCS), Avenida Central de Gandra 1317, Gandra, 4585-116 Paredes, Portugal; 5 REQUIMTE-LAQV, Departamento de Engenharia Metalúrgica e Materiais, Faculdade de Engenharia, UP, Rua Dr. Roberto Frias, 4200-465 Porto, Portugal; jdsantos@fe.up.pt; 6 Centre for Rapid and Sustainable Product Development (CDRSP), Polytechnic Institute of Leiria, Rua de Portugal—Zona Industrial, 2430-028 Marinha Grande, Portugal; nuno.alves@ipleiria.pt

**Keywords:** 3D printing, additive manufacturing, biomaterials, biomedical applications, bioprinting, cellular therapies, tissue regeneration

## Abstract

The application of three-dimensional (3D) printing/bioprinting technologies and cell therapies has garnered significant attention due to their potential in the field of regenerative medicine. This paper aims to provide a comprehensive overview of 3D printing/bioprinting technology and cell therapies, highlighting their results in diverse medical applications, while also discussing the capabilities and limitations of their combined use. The synergistic combination of 3D printing and cellular therapies has been recognised as a promising and innovative approach, and it is expected that these technologies will progressively assume a crucial role in the treatment of various diseases and conditions in the foreseeable future. This review concludes with a forward-looking perspective on the future impact of these technologies, highlighting their potential to revolutionize regenerative medicine through enhanced tissue repair and organ replacement strategies.

## 1. Introduction

Nowadays, regenerative medicine is considered an emerging field of research worldwide, with the potential to revolutionise healthcare (improving patient outcomes and quality of life) in the 21st century [1]. This field is focused on the replacement, engineering and regeneration of human cells, tissues and organs. Its aim is to repair, restore, supplement or replace the normal function of a biological system following treatment with autologous, allogeneic stem and stromal cells [2,3,4].

In recent years, two promising approaches have gained attention: namely, three-dimensional (3D) printing and cellular therapies. 3D printing has revolutionized tissue engineering through the manufacture of complex, patient-specific materials whilst providing precise control over the spatial distribution of cells and biomaterials. On the other hand, cellular therapies encompass the use of living cells to replace, repair, or regenerate injured tissues. The integration of 3D printing with cell therapies provides personalised, cell-laden constructs that mimic the complex architecture and function of native tissues. This approach stimulates cell–cell interactions, cell–matrix interactions and the creation of functional tissue-like constructs [5,6].

This review presents an overview of the current advances in the combination of 3D printing/bioprinting and cell therapies in the field of regenerative medicine. Firstly, the principles and applications of 3D printing/bioprinting and cell therapies are addressed individually. Subsequently, we explore recent advances in the integration of these technologies to create 3D-printed cellular structures. The limitations and challenges associated with these approaches are also mentioned, and the current state of the field is analysed. Finally, we present a future perspective on the potential impact of these technologies in the field of regenerative medicine and conclude with a summary of the main findings.

By providing a detailed understanding of the current state of the field and future directions, this review seeks to contribute to the growing body of knowledge in this challenging and rapidly evolving field.

## 2. Three-Dimensional Printing

Three-dimensional (3D) printing (alternatively termed additive manufacturing (AM)) is a burgeoning technique for the rapid prototyping of structures in diverse fields, including regenerative medicine [7,8,9,10,11]. In the past two decades, 3D printing has been widely researched due to its simplicity and the fact that its highly flexible manufacturing provides unlimited possibilities for creating complex structures [12,13]. Several examples of 3D printed medical devices include the following: instrumentation, including guides to facilitate the correct surgical placement of devices; implants, such as hip joints; and external prostheses, such as bionic hands [14].

**Figure 1:**
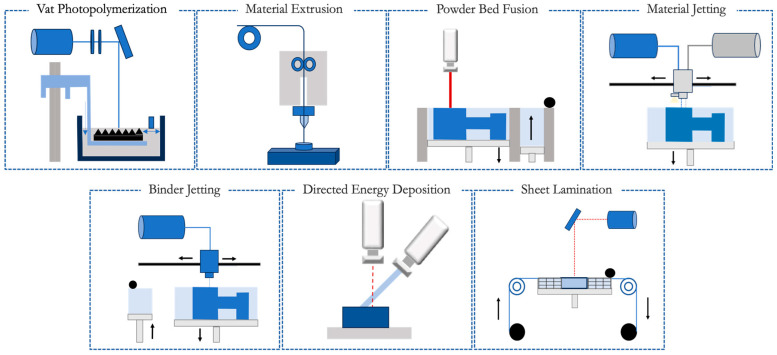
Schematic representation of the various AM processes. The black arrows in the figure illustrate the trajectory of the machine.

This advanced manufacturing technology, pioneered by Charles Hull in the early 1980s, enables the creation of customized and intricate structures with high precision and accuracy [15]. The technology operates by using computer-aided design (CAD) software to create a virtual model of the structure, which is subsequently converted into a physical object through the use of various 3D printing techniques [16]. The International Standard Organization (ISO) and the American Society for Testing and Materials (ASTM) [17] have established a classification system comprising seven overarching categories to categorize AM methods based on part manufacturing approaches, as outlined in Figure 1. The subsequent sections provide a succinct overview of AM processes.

### 2.1. Vat Photopolymerization

This method is characterized by the principle of selectively solidifying a liquid photopolymer resin by exposing it to specific light sources or patterns. In the procedure, a vat contains the liquid resin, which is selectively cured layer by layer in the desired regions, gradually constructing the intended object. The laser, guided by predetermined digital coordinates, provides the necessary information to cure the resin at precise locations within each layer. To facilitate this curing process, a photoinitiator is introduced into the resin material. The photoinitiator absorbs the incident ultraviolet (UV) radiation and generates active species that initiate the photopolymerization reaction [18,19].

The vat photopolymerization technique includes various commonly utilised processes, such as stereolithography (SLA), continuous light interface production (CLIP), digital light processing (DLP) and two-photon polymerization (2PP) [20]. These methods involve SLA, which uses a laser or digital light projector to cure the resin layer by layer, whereas DLP employs a digital light projector to cure an entire layer at once. CLIP employs a continuous liquid interface production approach, in which a continuous liquid interface is established between the resin and a transparent window, that enables continuous printing. Lastly, 2PP utilises a focused laser beam and a photosensitive resin to achieve high-resolution printing via a nonlinear optical process [21]. These vat photopolymerization techniques offer versatile and AM capabilities for fabricating complex and detailed structures.

### 2.2. Material Extrusion

Materials extrusion is a widely adopted AM technique due to its rapid manufacturing, cost efficiency, simplicity, user-friendly nature and the potential to produce complex components [22,23,24]. This technique involves extruding the material through an orifice and depositing it onto a construction platform [25,26,27].

The most common technique is fused filament fabrication (FFF), which is also referred to as fused deposition modelling (FDM) [28]. In this method, the filament is heated to a temperature typically between 150 and 250°C until it melts, and the molten material is extruded through a nozzle [25]. The extruded material is then placed onto a build plate, which can be adjusted in the z direction. This technique usually uses more polymers, although it is also used to print metal and ceramic components. FFF uses a support to create overhang features, which can be removed either mechanically, by detaching it from the printed part, or chemically, by dissolving it in a solvent [29]. In contrast to FFF, which employs thermoplastics, direct ink writing (DIW) is a 3D printing technique that utilises viscoelastic inks. In the DIW process, these inks are extruded through a nozzle mounted on a 3D translation platform, with the flow regulated by the application of pressure [30].

### 2.3. Powder Bed Fusion

The powder bed fusion (PBF) technique uses a laser or an electron beam to melt and fuse the material into powder [31]. The principle used in this technique is to produce the product layer by layer and melt it. A heat source concentrates its heat on a powdered base material and heats the cross-sectional area [32].

PBD is mainly used due to the low cost of producing the object and because the powder can be recycled to produce another piece [33]. This method encompasses several commonly utilised processes, including direct metal laser sintering (DMLS), selective laser sintering (SLS), electron beam melting (EBM), and selective laser melting (SLM). PBF utilises a laser source or an electron beam (EBM) to selectively melt or sinter layers of material, thereby fabricating a solid component. This technique is applicable to a range of powder-based materials, with metals and polymers being the most commonly processed [29].

### 2.4. Material Jetting

The material jetting (MJ) deposits the liquid in droplets to bind powder material [34]. In the MJ, the material is injected into the surface/building platform, where it solidifies, and the model is assembled layer by layer. The layers are subsequently cured or toughened with ultraviolet light. In this method, the material must be deposited in droplets, and therefore the materials used are limited. Generally, polymers and waxes are used, considering their viscous nature and ability to produce droplets [35]. This is a fast and proficient method and offers greater freedom when designing and printing complex models [24].

### 2.5. Binder Jetting

In binder jetting (BJ), a liquid bonding compound is applied selectively to bind powder materials. A relevant feature of this technique today is the possibility of using colour inkjet technology to create coloured objects in the binders [36].

Binders are used to ensure adhesion between the powdered material particles. These binders contribute to obtaining the strength of the part and the desired form of the final product [32,37]. The materials commonly used in this method are metals (e.g., stainless steel), ceramics (e.g., glass) and polymers (e.g., acrylonitrile butadiene styrene, polyamide and polycarbonate) [33].

### 2.6. Directed Energy Deposition

Directed energy deposition (DED) is an AM technique that utilises focused thermal energy to melt materials, which are fused as they are deposited. In this method, an energy source—such as an electron beam, a laser or a plasma—is used to melt the materials, which are then deposited [36].

The DED involves a nozzle assembled on a multi-hub arm that deposits the dissolved material onto a surface layer, where it solidifies. A significant advantage of this method is its ability to precisely control the grain structure of the deposited material [33,38,39].

This process is analogous to material extrusion; however, the nozzle in DED has the capacity to transverse multiple directions rather than being restricted to a single axis. While the process is applicable to polymers and ceramics, it is predominantly utilised for metals, which are provided in either powder or wire form [40].

### 2.7. Sheet Lamination

Sheet lamination involves the sequential bonding of thin sheets of material, normally fed through a set of rollers [41]. This technique can employ various materials, including paper, polymer and metal [28]. Although this is a less precise method, it offers advantages in terms of speed and cost-effectiveness [41].

The predominant sheet lamination techniques are laminated object manufacturing (LOM) and ultrasonic additive manufacturing (UAM). LOM employs a layer-by-layer method similar to other additive manufacturing processes, utilizing paper as the primary material and adhesive for bonding, rather than welding. Conversely, UAM involves metals such as aluminium, stainless steel and titanium, and operates at relatively low temperatures, enabling the production of complex internal geometries [42].

To highlight the advantages and drawbacks of each of the previously discussed AM techniques, a summary is provided in Table 1.

## 3. Bioprinting

According to Moroni et al., in the context of tissue engineering and regenerative medicine, the term “bioprinting” refers to the use of computer-assisted transfer techniques for the modelling and assembly of living and non-living materials in a predefined 2D or 3D configuration to create bioengineered structures [45]. By incorporating active substances, such as biomaterials, living cells and bioactive molecules, bioprinting imparts biological functions to the manufactured structures [12].

Bioprinting technology allows for the manufacture of complex, functional structures that promote cell growth and tissue formation. The prospect of manufacturing complete tissues or organs using 3D printing is very promising and has the potential to revolutionise regenerative medicine [46].

**Figure 2:**
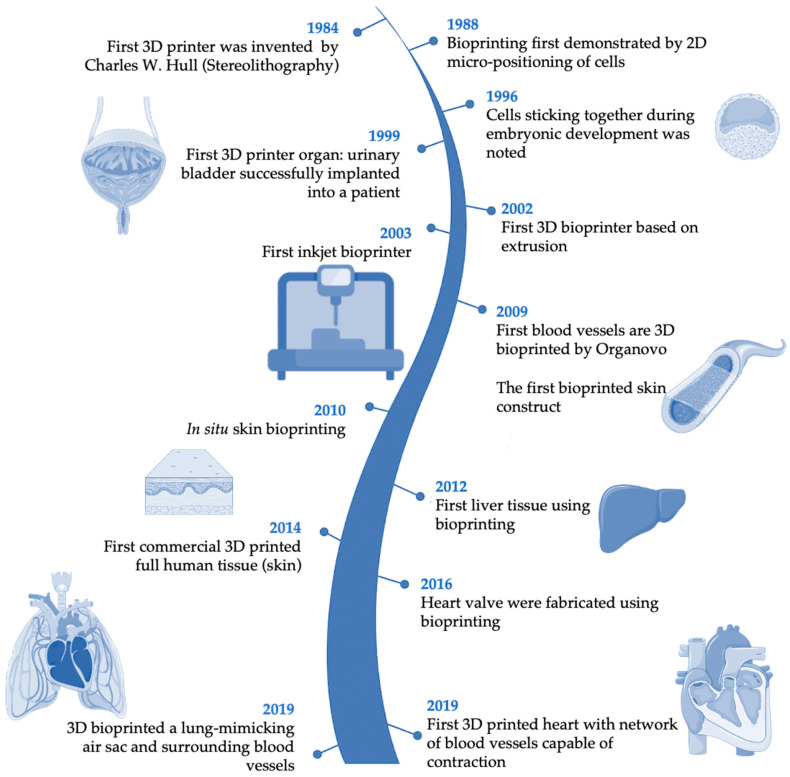
Major milestones in the history of 3D bioprinting.

In recent decades, significant advancements have been made in the field of 3D bioprinting (Figure 2) [47,48,49,50]. The evolution of this technology began with the invention of SLA by Charles Hull in 1984, which marked the beginning of 3D bioprinting [48]. Subsequently, in 2002, Landers et al. introduced extrusion-based bioprinting technology, which was later commercialized under the name “3D-Bioplotter” [51,52]. In 2003, Thomas and Boland’s research team adapted a conventional inkjet printer to develop the first inkjet bioprinter capable of printing living cells [51,52]. Later, the engineering of scaffold-free vascular tissue via bioprinting was achieved by Norotte et al. [53]. The subsequent years witnessed the development and introduction of various bioprinted constructs, including an artificial liver in 2012, full human skin in 2014, a heart valve in 2016, and a lung-mimicking air sac with surrounding blood vessels in 2019, among other innovations [47,48,49,50].

### 3.1. Techniques

Among the diverse AM techniques, the most employed in bioprinting are laser-based printing (SLA and SLS), extrusion printing and inkjet printing [54,55,56].

SLA is a vat photopolymerization process that consists of photocurable bioinks that are subjected to UV, infrared or visible light to produce 3D pieces using the layer-by-layer procedure [57,58]. Among the advantages of SLA is its capacity to rapidly cure at physiological temperatures, which facilitates the production of constructs suitable for regenerative medicine applications. This technique has been widely employed to fabricate micro-needles designed for transdermal drug delivery, hearing aids, surgical guides for placing dental implants, temporary crowns and bridges and supports for tissue engineering with/without encapsulated cells [57,59].

During SLS printing, fine particles of the entire substance are fused by the heat of a high-powered laser to fabricate a 3D structure [60,61]. In this technique, several categories of powders, including polymers, ceramics and metals, must be processed into powder. SLS is a PBF process that is used in numerous applications in the medical field, namely the fabrication of prototypes for medical devices, physical models used in surgery and scaffolds for tissue engineering. The popularity of SLS printers is attributed to their affordability, high productivity and material versatility [61,62].

Extrusion printing is considered the most popular bioprinting technology [63]. In this method, material is melted and extruded through a nozzle, orifice or needle using a screw, piston or high-pressure pneumatic force to form successive layers of the part [64,65,66,67,68]. This technique has been used extensively in the medical field, enabling the biofabrication of tissues, organs, implants and personalised drug delivery systems. Extrusion is also highly applicable in the field of disease modelling. Models made by extrusion can provide a baseline for the comprehension of the underlying biological mechanisms behind disease progression, thus contributing to the identification of effective treatments [69].

Inkjet bioprinting is considered the pioneering technology in the field of bioprinting. The printing process utilizing this technique comprises two phases: first, the generation of discrete droplets that are precisely directed to specific locations on a substrate, and second, the subsequent interaction between these droplets and the substrate [48,70]. It is employed in various medical applications, including the creation of patient-specific or project-specific implants for static load-bearing purposes (such as dental crowns and prosthetic structures), joint applications (such as osteochondral cartilage implants) and for facilitating in vivo blood vessel formation and tissue regeneration [71].

Table 2 presents the aforementioned techniques, as well as the advantages and drawbacks of each.

### 3.2. Materials Used in 3D Bioprinting

Nowadays, a diverse range of biomaterials are being used in 3D bioprinting [76]. The interaction between biomaterials and cells is fundamental for cell viability, proliferation, and differentiation. Hence, it is fundamental to consider the characteristics of biomaterials, such as non-toxicity, biocompatibility, and the absence of immune reactions and foreign body responses [77].

Bioprinting materials are categorized into two types: natural and synthetic biomaterials. Natural biomaterials are particularly attractive due to their bioactivity, being both similar to the extracellular matrix (ECM) and biocompatible. The following are some examples of natural materials used in this field: collagen, xanthan gum (XG), silk fibroin (SF), gelatin, pectin, gellan gum, albumin, chitosan, sodium alginate, agarose, fibrin, keratin and hyaluronic acid (HA). However, these types of materials generally exhibit poor mechanical properties [78,79,80,81,82,83,84,85,86,87,88,89,90,91,92,93,94,95,96,97,98,99]. Conversely, synthetic materials provide distinct advantages compared to natural materials, including the ability to be customized with specific physical properties and greater uniformity. The following are some examples of synthetic materials used in this field: poly (ℇ-caprolactone) (PCL), poly(lactic-co-glycolic) acid (PLGA), poly(l-lactic) acid (PLA), poly (glycolic acid) (PGA), polyurethane (PU), polyethylene glycol (PEG), polyether ether ketone (PEEK), polyvinylpyrrolidone (PVP) and pluronic. Nevertheless, synthetic materials for 3D bioprinting have some disadvantages, such as poor biocompatibility, the potential release of toxic degradation byproducts and the absence of bioactive ligands [100,101,102,103,104,105,106,107,108,109,110,111,112,113].

To address these drawbacks, a more comprehensive knowledge of the physiological characteristics of the ECM and a higher ability to replicate the complex 3D structures mimetic of the ECM would represent a significant advance in 3D bioprinting. Advances in composite and hybrid bioprinting materials, as well as multimaterial bioprinting technologies, have emerged in the field of tissue regeneration [114,115]. Nonetheless, human tissues and organs operate within a highly dynamic biochemical environment, and conventional bioprinted materials often fail to adapt to the evolving spatial and temporal requirements of tissue and organ development. Consequently, there is increasing interest in developing sophisticated stimuli-responsive biomaterials for 3D bioprinting, which would facilitate the adaptation of artificial constructs to intricate and dynamic physiological conditions [115,116,117].

## 4. Cellular Therapies

Cellular therapies, also known as cell therapy, cell transplantation or cytotherapy, involve the injection, grafting or implantation of cells into a patient (autologous or allogeneic) to achieve a medicinal effect [118,119]. Cell therapies are frequently applied in combination with biomaterial supports, which are designed to support and guide the cells both during and after transplantation [120].

Advances in 3D printing technology allow for the precise spatial arrangement of multiple cells and biomaterials, thereby enhancing the efficiency of cell delivery and integration onto scaffolds. There are two primary methods to deliver cells via 3D printed scaffolds: post-printing cell seeding and embedding cells within bioinks during the 3D bioprinting process [121,122,123,124]. The approach of seeding the cells after printing provides more flexible requirements for both the conditions and 3D printing process, though it is hindered by low cell adhesion rates on the scaffolds. To address this challenge, one approach involves encapsulating cells in hydrogels prior to combining them with 3D printed scaffolds. Alternatively, the bioprinting method enhances cell loading efficiency and allows for precise control over the spatial distribution of various cell types. However, this method demands stringent conditions and precise parameters during printing, as cells are sensitive to suboptimal environmental conditions. This necessitates detailed knowledge of the parameters of the bioinks and printing conditions beforehand [74,125,126,127,128].

The selection of a specific type of cell therapy is highly important, since it affects the function and design of the tissue engineering model [129,130]. There are three categories of cell therapies that can be applied to printed scaffolds, namely, stem cell-based, non-stem cell-based and multicellular therapies [118].

### 4.1. Stem Cell-Based Therapies

Stem cells are among the most frequently utilised cell types due to their unspecialized nature, which allows them to self-renew and differentiate into various cell lineages [11,129,131,132]. Their ability to differentiate into specific cell types while continuously dividing and self-renewing makes their prospects interesting for regenerative medicine. They have successfully been used to create functional tissues that replicate the properties of natural organs. These stem cells can be sourced from several origins [133]. In the field of bioengineering, the three most frequently employed types are mesenchymal stem/stromal cells (MSCs), embryonic stem cells (ESCs) and induced pluripotent stem cells (iPSCs) [134].

Also known as multipotent cells, MSCs can be sourced from various tissues, including bone marrow, muscle, lung, teeth, adipose tissue, liver and perinatal/extra-embryonic tissues. They exhibit the capacity to proliferate and differentiate into a broad range of cell types, such as osteoblasts, chondrocytes and adipocytes [135]. Additionally, MSCs can differentiate into other mesenchymal and non-mesenchymal cells, including myocytes, tendocytes, neural cells, ligament cells, smooth muscle cells, endothelial cells, cardiomyocytes and hepatocytes [135,136]. In regenerative medicine, these cells offer numerous advantages, including ease of expansion in culture and the capacity to differentiate into desired cell lines. They also possess specific immunological properties, such as being immunoprivileged and immunomodulatory, and have tropisms for injury sites. Additionally, they can stimulate trophic responses and modulate tissue functions and inflammation through the secretion of essential bioactive molecules [137]. However, MSCs present some possible challenges, such as poor-quality control and inconsistencies regarding their heterogeneity, stability, differentiation, immunocompatibility, and migratory capacity [138,139].

In turn, ESCs are pluripotent cells originating from the inner cell mass of blastocytes (an early embryo). They possess the capability to differentiate into nearly all cell types originating from the three germ layers, with the exception of trophoblastic cells [140]. However, ESCs raise ethical concerns, exhibit challenges in controlling differentiation, and have the potential to form teratomas and provoke immune responses [134,141,142,143,144].

Finally, iPSCs are pluripotent stem cells generated by reprogramming adult somatic cells through the introduction of specific genes and factors, thereby mimicking the pluripotent state of ESCs [145]. While iPSCs share many characteristics with ESCs, they do not carry the same ethical issues or immunogenicity concerns. Nonetheless, iPSCs, such as ESCs, are also associated with the risk of teratoma formation in vivo [134].

### 4.2. Non-Stem Cell-Based Therapies

Non-stem cell therapies commonly use somatic cells that are isolated from humans, subsequently cultured and expanded in vitro and then applied to patients for therapeutic, preventive or diagnostic treatment [146]. These can be categorized into immune cells and non-immune cells. Immune cells, including natural killer cells, dendritic cells and macrophages, can be engineered to target specific antigens. This approach is used in therapeutic strategies, including for cancer, infections, autoimmune diseases and allogeneic transplantation. On the other hand, non-immune cells, such as chondrocytes, fibroblasts, hepatocytes, keratinocytes and pancreatic islet cells, normally are involved in the host’s defence response as structural architectures, regulators and effectors of its protective immune reaction [118,146,147,148,149,150].

These somatic cell-based therapies serve as in vivo resource of cytokines, enzymes and growth factors. Additionally, they are frequently used in adoptive cell therapy for cancer treatment and as transplanted cells (e.g., hepatocytes or pancreatic islet cells) to address genetic metabolic disorders. They also find applications as cell-based or scaffold-free systems in the treatment of burns, ulcers and cartilage injuries [118,147,151].

### 4.3. Multicellular Therapies

Multicellular therapies comprise at least two types of cultured stem and/or non-stem cells. This emerging approach holds that using a combination of cell types is more effective for promoting long-term tissue repair compared to single-cell therapy. This effectiveness is attributed to cell–cell interactions that extend beyond embryogenesis and play a crucial role in regenerative procedures [118,149,152].

Multicellular therapies include scaffold-supported and scaffold-free cell products, bone marrow aspirate-derived therapies, adoptive cell therapy products, stem cell transplantation and stromal vascular fraction [118,147,153,154,155].

## 5. Advancements in 3D Printed/Bioprinting and Cellular Therapies for Regenerative Medicine

Advancements in combining 3D printing/bioprinting and cellular therapies hold considerable promise for advancing regenerative medicine. This synergy has the potential to enhance tissue engineering capabilities and introduce novel therapeutic approaches for a range of diseases and conditions. Such advancements could substantially improve patient outcomes and have the potential to significantly impact survival rates [13,156,157].

**Figure 3:**
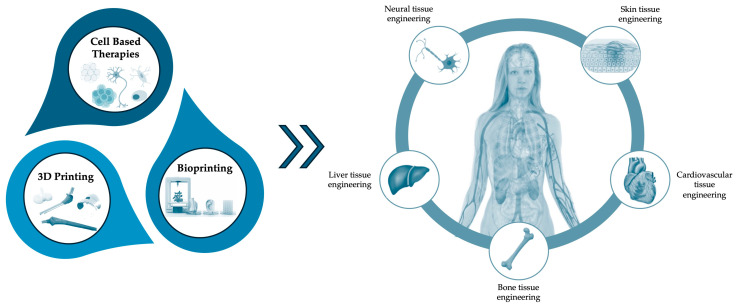
Advances in cell therapies, 3D printing and bioprinting are addressing the challenges in engineering cardiovascular, bone, liver, skin and neural tissues.

This approach has been successful in printing the structures of different tissues, including cardiovascular, bone, liver, skin and neural tissues (Figure 3). The following sections analyse the advancements in the integration of 3D printing/bioprinting and cellular therapies in several regenerative medicine applications.

**Figure 4:**
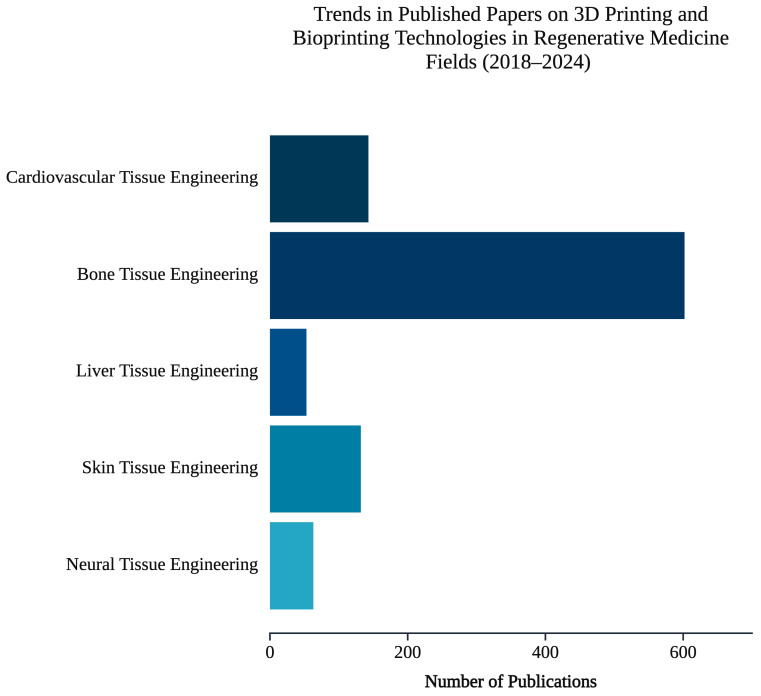
Trends in published papers on 3D printing and bioprinting technologies across regenerative medicine fields: cardiovascular, bone, liver, skin and neural tissue engineering. The number of publications was analysed using the PubMed database from 2018 to 2024, employing the search terms: (“Cardiovascular”)/(“Bone”)/(“Liver”)/(“Skin”)/(“Neural”) AND ((“3D Printing”) OR (“Bioprinting”)).

Over recent years, research concerning 3D printing and bioprinting technologies in regenerative medicine has grown significantly. As indicated in Figure 4, the number of publications related to bone regeneration is noticeably higher than the other fields. This prominence can be attributed to the compatibility of bone with 3D printing technologies, which allow precise control of scaffold porosity and the development of patient-specific implants. Furthermore, the high clinical demand for bone regeneration solutions, the limitations of traditional grafting techniques, and advancements in clinical translation, including regulatory approvals of 3D-printed bone supports, have significantly contributed to the extensive research focus in this area [158,159,160].

### 5.1. Cardiovascular Tissue Engineering

Cardiovascular diseases, which include pathologies affecting the myocardium, heart valves and body’s vasculature, are highly prevalent worldwide. These diseases are a leading cause of morbidity and mortality, especially in developed countries [161,162,163,164]. Current therapeutic approaches include cellular therapies, bypass grafting, the implantation of medical devices, cardiac tissue patches, and organ transplantation [161,165,166,167,168]. Organ transplantation is often not the optimal solution due to the imbalance between the availability of donor organs and the high demand. Additionally, the success of organ transplants is frequently compromised by complications related to immune rejection [162,169]. Among the various therapeutic approaches, cell therapy has demonstrated success in regenerating cardiovascular tissue. Nevertheless, the absence of ECM limits cell survival following injection, resulting in reduced long-term viability [169].

To address these concerns, 3D printing and bioprinting have emerged as effective approaches for developing scaffolds that incorporate ECM components and enhance cell viability [170]. These structures can more accurately mimic the spatial and mechanical properties of native tissues, which is relevant for their functionality and integration in vivo [164]. Currently, scaffolds for cardiovascular tissue engineering have been fabricated using various 3D printing technologies, such as inkjet printing, SLA and extrusion-based techniques.

These scaffolds commonly incorporate biocompatible materials of natural origin, including fibrin, alginate, gelatin, collagen, HA and fibrinogen [171]. However, to achieve complex structures with optimal physical, chemical and mechanical properties, synthetic materials, including poly (glycerol sebacate) (PGS), PCL and poly (ethylene glycol) methacrylate (PEGMA), are also employed. Additionally, some studies utilise a combination of natural and synthetic materials or semi-synthetic derivatives, such as gelatin methacrylate (GelMA) and hyaluronic acid-gelatin methacrylate (HAGM), to optimize scaffold properties while maintaining cell viability [13]. Furthermore, decellularized matrices are extensively utilised due to their provision of a porous, interconnected polymeric network that facilitates cell migration, proliferation and the delivery of essential nutrients for cell survival [172].

**Figure 5:**
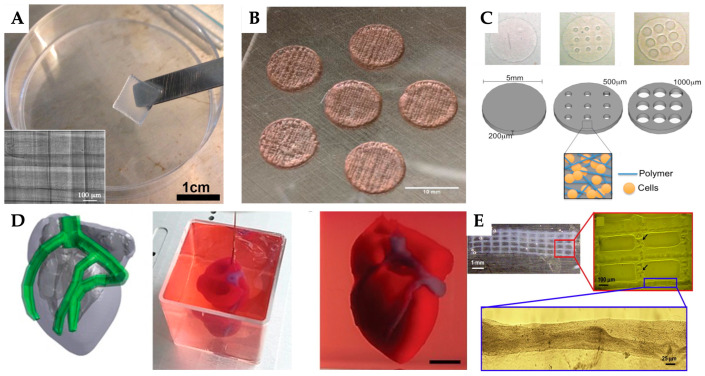
Advances in 3D printing/bioprinting and cell therapies in cardiovascular engineering: (**A**): Bioprinted cellularised construct incorporating human Umbilical Vein Endothelial Cells (hUVECs) and induced pluripotent stem cell-derived cardiomyocytes (iPSC-CMs), embedded in alginate and PEG-Fibrinogen (PF) (adapted from [173]); (**B**): Printed patches composed of decellularized cardiac extracellular (cECM) matrix hydrogel and GelMA, for delivery of paediatric human cardiac progenitor cells (hCPCs) (adapted with permission from [174], Copyright 2018 John Wiley and Sons); (**C**): MSC-loaded gel patches with microchannels of controlled diameters. Three models tested the following: one without microchannels and two with nine evenly spaced microchannels, maintained at diameters of 500 μm and 1000 μm (reprinted (adapted) with permission from [175]. Copyright 2017, American Chemical Society); (**D**): A 3D-printed cellularised human heart is shown. (Left: CAD model of the human heart; middle and right: the printed cardiac structure immersed in a supportive bath) (adapted from [176]); (**E**): Printed fibrin scaffold using modified thermal inkjet printer (adapted with permission from [177], Copyright 2009 Elsevier).

Maiullari et al. developed a technique for fabricating 3D cardiac tissue models that include a vascular network (Figure 5A). They created multi-cellular constructs using human umbilical vein endothelial cells (hUVECs) and induced pluripotent stem cell-derived cardiomyocytes (iPSC-CMs), encapsulated in alginate and PEG-Fibrinogen (PF). The constructs were fabricated using a custom-designed microfluidic printhead operating at an extrusion speed of 180 mm/min. Their research demonstrated that bioprinted endothelial cells are capable of forming functional vascular structures within the transplanted tissues and interacting with the host’s existing vascular network [173].

To address damaged myocardium, Beijleri et al., produced a 3D-printed cardiac patch consisting of a decellularized cardiac extracellular matrix (cECM) hydrogel combined with GelMA. This patch was designed for the delivery of paediatric human cardiac progenitor cells (hCPC) (Figure 5B). The GelMA-cECM bioinks ensure uniform distribution of cECM and hCPCs, with the hCPCs maintaining over 75% viability. Additionally, conditioned media from GelMA-cECM patches demonstrate more than a 2-fold increase in angiogenic potential. The patches also remain adhered to rat hearts and show vascularization over a 14-day period in vivo [174]. Also, in the development of tissue patches, Melhem et al. proposed a hydrogel patch embedded with multiple microchannels to enhance cell retention and factor delivery at the target tissue (Figure 5C). They integrated bone marrow-derived MSCs (BMSCs) into a hydrogel by cross-linking a poly (ethylene glycol) dimethacrylate (PEGDMA) solution containing the cells. Microchannels with precise diameters were created within the cell-loaded hydrogel using an SLA unit for in situ cross-linking. This 3D-printed, microchanneled hydrogel was designed as an advanced therapeutic tool for sustained delivery of multiple therapeutics, aiming to improve outcomes in ischemic heart injury [175].

Noor et al. established a technique to 3D print thick, vascularized cardiac patches tailored to a patient’s specific properties. They used patient-derived cells, reprogrammed into cardiomyocytes and endothelial cells, and a personalized hydrogel made from the patient’s extracellular matrix. These components were combined to create bioinks for printing cardiac tissue and blood vessels. The patches, optimized for oxygen transfer, demonstrated proper structure and function in vitro, and the approach was further validated by successfully printing cellularised human hearts presenting a natural architecture (Figure 5D) [176].

In turn, to mimic human microvasculature, Cui et al. developed a bioink combining human microvascular endothelial cells (hMVECs) and fibrin, used to fabricate micron-sized fibrin channels via drop-on-demand polymerization (Figure 5E). This aqueous-based printing method reduces cellular damage. The hMVECs printed with fibrin aligned within the channels and proliferated to form continuous linings, resulting in a three-dimensional tubular structure. The study concluded that the concurrent printing of cells and scaffolds enhances the proliferation of hMVECs and supports the formation of microvascular networks [177].

Several researchers have employed 3D printing or bioprinting, with or without cell therapies, to advance cardiovascular tissue engineering. Table 3 provides a summary of these studies.

### 5.2. Bone Tissue Engineering

Severe bone defects resulting from aging, trauma, osteoporosis, degenerative diseases, autoimmune diseases (such as rheumatoid arthritis) or tumour removal are a leading cause of disability globally, affecting an estimated 1.71 billion people [189,190]. Current therapeutic strategies for addressing these defects include autografting, allografting, xenografting and bone transplantation. Despite their application, these methods present significant risks, including the potential for infectious disease transmission and immune rejection [191,192]. To address the limitations of traditional bone defect treatments, 3D printing and bioprinting techniques, with or without cellular therapies, have been developed for bone tissue engineering. These methods enable the large-scale fabrication of custom-tailored bone tissues, meeting the growing demand for functionalized bone implants [193]. The 3D-printed scaffolds serve as a support for cell growth and differentiation, forming a hierarchical bone microvascular architecture [194]. For optimal performance, a scaffold should possess several critical attributes: biocompatibility, sterility, osteoconductivity, biodegradability and a porous, interconnected structure that supports cellular infiltration and nutrient transport. Additionally, it should effectively repair bone defects while closely mimicking the characteristics of native bone tissue [16,195,196].

The most commonly employed printing techniques in bone tissue engineering are laser powder bed fusion, vat photopolymerization and extrusion-based methods.

Various biomaterials are utilised for printing scaffolds: ceramics (e.g., beta-tricalcium phosphate (β-TCP), hydroxyapatite (HAp) and amorphous calcium phosphate (ACP)); natural polymers (e.g., matrigel, alginate, HA and dextran emulsion); synthetic polymers (e.g., GelMA, PLGA, PCL and polyethylene glycol diacrylate (PEGDA); metals (e.g., titanium alloy (Ti6Al4V), tantalum (Ta) and titanium (Ti)), as well as the combinations of these materials.

**Figure 6:**
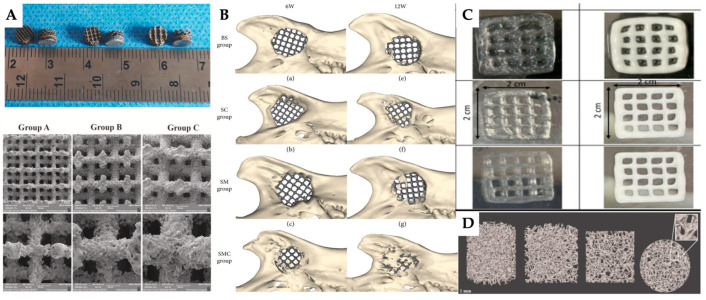
Advances in 3D printing/bioprinting and cell therapies in bone tissue engineering: (**A**): Ti6Al4V-based porous tantalum (Ta) scaffolds and scanning electron microscopy images (adapted from [197]); (**B**): Newly formed bone within the scaffold was assessed through ex vivo micro-computed tomography(micro-CT) scans at 6 and 12 weeks (adapted from [198]); (**C**): Visual representations of 3D-printed samples at different alginate concentrations, alginate alone or alginate with beta-tricalcium phosphate (β-TCP) (adapted with permission from [199], Copyright 2024 Elsevier); (**D**): Bone scaffolds based on magnesium (Mg^2+^), strontium (Sr^2+^) and zinc (Zn^2+^)-substituted hydroxyapatite, as represented in the CAD model [200]).

Lei et al. developed Ti6Al4V-based porous tantalum (Ta) scaffolds with high interfacial strength using laser powder bed fusion. In this process, porous Ta was directly deposited onto a solid Ti6Al4V substrate (Figure 6A). The manufacturing process was carried out in an argon atmosphere, using a 300 W laser at 250 mm/s for Ta and a 250 W laser at 300 mm/s for Ti6Al4V. In vitro biocompatibility assessments conducted with rat bone marrow mesenchymal stem cells (r-BMSCs) confirmed the scaffolds’ biocompatibility. The findings demonstrated the strong mechanical compatibility and osteointegration properties of the Ti6Al4V-based porous Ta scaffold, underscoring its considerable potential for orthopaedic applications [197].

For mandibular bone defect reconstruction, Yu et al. encapsulated BMSCs in matrigel and infiltrated this mixture into porous Ti6Al4V scaffolds. The study demonstrated that rats with critical full-thickness mandibular defects treated with Matrigel-infused Ti6Al4V scaffolds exhibited significantly greater new bone formation compared to those treated with either local BMSC injections or Matrigel alone (Figure 6B). These results indicate that Matrigel enhances the 3D microenvironment for BMSCs, positioning Matrigel-infused scaffolds as a promising method for improving bone regeneration in 3D-printed Ti6Al4V scaffolds [198].

Wu et al. produced a 3D-bioprintable scaffold combining alginate and β-TCP for the treatment of bone defects (Figure 6C). The scaffolds were fabricated at a printing speed of 2 mm/s, with an extrusion pressure ranging from 60 to 200 kPa. MG-63 cells were seeded onto these scaffolds. The 3D-printed scaffolds using a 10% alginate/β-TCP bioink exhibited enhanced physical characteristics and significantly improved cell viability and alkaline phosphatase activity. These findings suggest that the scaffolds have considerable potential for application in personalized bone regeneration therapies [199].

To produce a scaffold that replicates bone microstructure, Ressler et al. developed trabecular-like porous scaffolds using ceramic vat photopolymerization with HAp powders doped with magnesium (Mg^2+^), strontium (Sr^2+^) and zinc (Zn^2+^). Scaffolds sintered at 1100–1300 °C exhibited mechanical properties similar to trabecular bone, with optimal performance at 1300 °C. The microstructure resembled cancellous bone and the incorporation of trace elements resulted in a biphasic calcium phosphate system (HAp/β-TCP), potentially enhancing bioactivity [200].

Numerous studies have utilised 3D printing or bioprinting, either with or without cellular therapies, to advance bone tissue engineering. An overview of these studies is provided in Table 4.

### 5.3. Liver Tissue Engineering

The liver plays a vital role in blood protein synthesis, glucose metabolism and the detoxification of metabolites [214]. It is also the only organ in the human body capable of efficient regeneration. However, this regenerative capacity can be compromised by excessive drug use or viral infections, which can cause irreversible damage to hepatocytes and lead to liver failure [215,216]. Chronic liver diseases, including fibrosis, cirrhosis, chronic viral hepatitis and fatty liver disease, significantly contribute to global morbidity and mortality. Unfortunately, advancements in treatment options for these conditions remain limited [217,218].

The primary medical intervention for liver failure is partial or total liver transplantation. Nevertheless, this approach faces challenges, including limited donor availability, immune rejection and variable graft success rates. Alternative approaches in tissue engineering encompass bioartificial liver systems, which involve the in vitro creation of liver tissue to repair or replace damaged liver segments, as well as hepatocyte transplantation and cellular therapy methods [215]. There has been a continuous search for a reliable and reproducible source of hepatocytes, whether for liver regeneration therapy, seeding liver support devices or in vitro screening applications [216].

Numerous studies have investigated the 3D bioprinting of liver tissue utilizing either stem cells or immortalized hepatic cell lines [219,220]. Stem cells are particularly promising due to their ability to express hepatocyte-like phenotypes. In contrast, adult hepatocytes are scarce, challenging to isolate, exhibit poor propagation and experience rapid functional deterioration in vitro [221].

In liver tissue engineering, a variety of biomaterials are employed, categorized into natural and synthetic polymers [222]. Natural polymers, such as alginate, HA, collagen, cellulose nanocrystal (CNC) and gelatin, present the advantages of enhanced cell compatibility and ease of manipulation. Nevertheless, they are constrained by relatively weaker mechanical properties, limited availability and variable degradation rates. Consequently, synthetic polymers, such as PCL, have been developed to provide superior mechanical strength, flexibility, processability and adjustable degradability. Despite these advantages, synthetic polymers generally lack cell recognition and adhesion sites, resulting in reduced biocompatibility compared to natural polymers [128]. To mitigate these issues, a strategy of combining natural and synthetic polymers is employed to create effective bioinks for 3D bioprinting. Additionally, the liver decellularized extracellular matrix (dECM), sourced from animals, is frequently used to establish microenvironments for liver cells, offering cross-species tolerance and minimising the risk of immune rejection [223,224,225].

**Figure 7:**
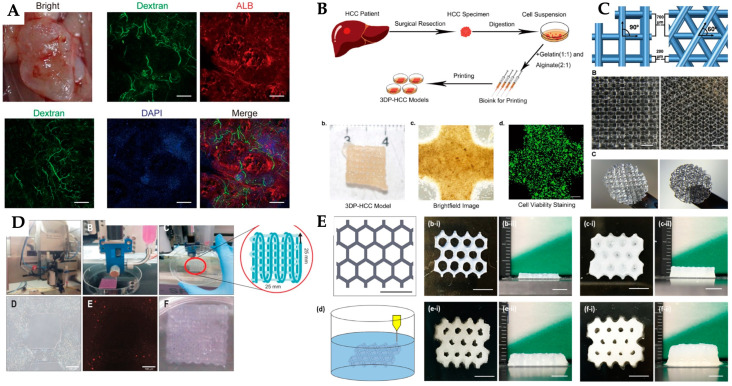
Advances 3D printing/bioprinting and cell therapies in liver tissue engineering: (**A**): Human liver functions of 3D bioprinted hepatorganoids in mice. First column: Macroscopic observation of transplanted 3D bioprinted hepatorganoids. The vascular system is illustrated following the infusion of dextran. Second and third column: The figure shows the dextran infusion with the formation of functional vessels and the red fluorescence showing the expression of human ALB at 4 weeks (adapted from [226]); (**B**): The study details the production of patient-derived 3D bioprinted hepatocellular carcinoma models. Initially, HCC samples were obtained following post-surgical resection and subsequently processed into cell suspensions. These suspensions were then combined with sodium alginate and gelatin to create the bioink used for bioprinting. The images provided in the second line include the following: (1) the general appearance of the 3D bioprinted hepatocellular carcinoma model, (2) a light microscopy image showing the distribution of cells within the model and (3) an in vitro view of the model, where live cells are marked in green and dead cells are marked in red (adapted with permission from [227], Copyright 2021 Elsevier); (**C**): First line: schematic representation of the 3D printed scaffold, including strut spacing, nozzle diameter and angle between adjacent layers (90 or 60 degrees). Second line: large-scale 3D printed gelatin structure. Third line: cross-linked scaffolds and biopsy-punched samples (adapted with permission from [228], Copyright 2018 Elsevier); (**D**): Construction of 3D-printed mCherry-HepG2 hepatic structures: (D-A) 3D bioprinting machine; (D-B) extrusion of alginate combined with mCherry-HepG2 cells through nozzle pressure; (D-C) layer-by-layer deposition of cross-linked structures in square arrays; (D-D) phase contrast microscopy showing a confluent monolayer of HepG2 cells; (D-E) fluorescence microscopy image of mCherry-HepG2 cells within the alginate scaffold; (D-F) multilayered mCherry-HepG2 cells solidified and stacked repetitively (adapted from [229]); (**E**): Schematic representation of the liver lobule-mimetic honeycomb structure, including top and side views of the embedded-printed scaffolds (adapted from [230]).

Yang et al. developed a liver tissue model through 3D bioprinting using HepaRG cells, a widely utilised hepatic progenitor cell line. Each 3D bioprinted hepatorganoid was produced through forced extrusion at a rate of 150 mm^3^/min, using a layer-by-layer approach. This 3D bioprinted model, referred to as hepatorganoids, exhibited essential liver functions, including albumin production, drug metabolism and glycogen accumulation, after a 7-day differentiation period (Figure 7A). In vivo studies showed that the 3D bioprinted hepatorganoids further matured, exhibiting enhanced synthesis of liver-specific proteins and more human-like drug metabolism. Notably, transplantation of 3D bioprinted hepatorganoids significantly increased survival rates in recipient mice. These findings suggest that 3D bioprinted hepatorganoids can effectively undergo hepatic differentiation and ameliorate liver failure in vivo [226].

In the study conducted by Xie et al., a 3D bioprinted model of hepatocellular carcinoma, derived from patient cells, was created using isolated primary hepatocellular carcinoma cells mixed with gelatin and sodium alginate to form a bioink (Figure 7B). The resulting models were successfully generated and exhibited substantial growth over prolonged culture durations. They preserved key characteristics of the original hepatocellular carcinoma tumours, including consistent biomarker expression and stable genetic alterations and expression profiles. Thus, 3D bioprinted hepatocellular carcinoma models prove to be reliable in in vitro systems, suitable for long-term culture and capable of predicting patient-specific drug responses for tailored therapeutic approaches [227].

Lewis et al. studied a technique for 3D printing gelatin into precisely defined geometries, which exhibit distinct biological effects on seeded hepatocytes (Figure 7C). Their research reveals that the structural configuration of gelatin can markedly impact biological processes. An undifferentiated hepatocyte cell line demonstrated high viability and proliferation on 3D-printed scaffolds with two distinct geometries. Notably, hepatocyte-specific functions—such as albumin secretion, cytochrome P450 activity and bile transport—were enhanced in more interconnected 3D-printed gelatin structures compared to those with less interconnectivity and traditional two-dimensional (2D) cultures. This study underscores the gap between gene expression and protein functionality in simplistic 2D cultures, highlighting the importance of a physiologically relevant 3D environment for optimizing hepatocyte expression and functionality [228].

Jeon et al. employed 3D bioprinting technology to reconstruct liver tissues and organs using human hepatocellular carcinoma (HepG2) cells, a liver cancer-derived cell line. They created multi-layered 3D structures by integrating alginate with HepG2 cells (Figure 7D). The study demonstrated that replicating the 3D hepatic architecture using this technology enhances the stability and gene expression profiles of HepG2 cells. Cells cultured on these 3D alginate scaffolds for three weeks were analysed via fluorescence microscopy, histology and immunohistochemistry. Results indicated that HepG2 cells exhibited improved growth and liver-specific gene expression in 3D cultures compared to 2D cultures, highlighting the effectiveness of 3D bioprinting in mimicking liver architecture and enhancing cellular function [229].

Using the same 3D bioprinting technique, Wu et al. produced a novel bioink containing alginate, CNC and GelMA (namely, 135ACG hybrid ink), aimed at fabricating both cell-laden and acellular structures (Figure 7E). The structures were printed using a 32-gauge nozzle at a printing speed of 20 mm/s and an air pressure of 20 psi. The bioink presented good shear-thinning behaviour and solid-like characteristics, ensuring high printability and minimal cell damage. Following crosslinking, it formed a rigid ECM conducive to stromal cell growth. The team engineered a GelMA bioink with suitable mechanical properties to mimic human liver tissue, enabling the printing of liver lobule-mimetic constructs with precise cell placement (fibroblasts and hepG2) in different ECMs (135ACG and GelMA). These constructs were used to study the impact of mechanical stimuli and cellular interactions on cell behaviour. These findings demonstrated that fibroblasts proliferated effectively within the rigid 135ACG matrix, whereas HepG2 cells developed into spheroid structures in the more compliant GelMA matrix. Co-cultures of hepG2 and fibroblasts cells showed increased albumin production, highlighting the role of soluble factors in enhancing hepatic function. The study demonstrated that the developed bioinks and printing methods are effective for creating complex, multi-cellular constructs with varied ECMs, advancing both fundamental research and tissue engineering applications [230].

Several studies have employed 3D printing/bioprinting with or without the incorporation of cellular therapies to advance liver tissue engineering. A summary of these studies is presented in Table 5.

### 5.4. Skin Tissue Engineering

The skin, regarded as the largest organ in the human body, plays essential roles in various functions, including serving as a protective barrier, regulating body temperature and preventing dehydration [240]. Extensive full-thickness skin wounds, which damage underlying blood vessels, pose serious risks due to induced cellular hypoxia and nutrient deprivation [241]. While autografts are considered the “gold standard” for treating severe skin injuries, their use is limited by issues such as donor site availability and associated morbidity [242]. Furthermore, existing commercial skin substitutes lack the sufficient vascular networks necessary for effective nutrient delivery in full-thickness wounds. Consequently, skin substitutes are seen as promising alternatives, offering the potential for vascularized skin reconstruction with customized cell compositions and controlled geometrical structures [241,243].

**Figure 8:**
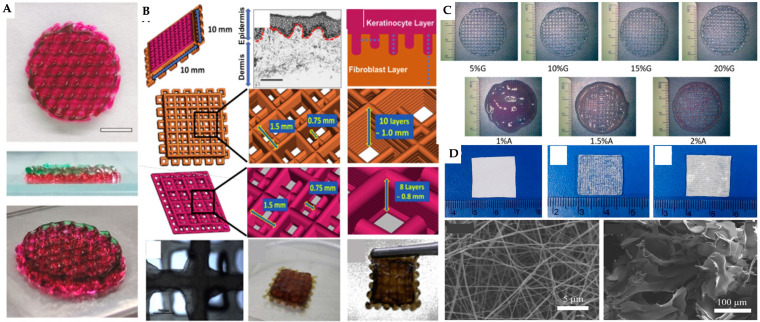
Advances 3D printing/bioprinting and cell therapies in skin tissue engineering: (**A**): The image shows the printed epidermal layer (red) composed of alginate-gelatin hydrogel, and the dermal layer (green) made of phosphosilicate calcium bioglass (PSC)-alginate-GelMA hydrogel embedded with hUVECs and hUCMSCs (adapted with permission from [241], Copyright 2024 Elsevier); (**B**): Diagram overview of the produced structure design: diagram illustration of the CAD model; illustration of human skin anatomy, with the dermis and epidermis; approach for designing the dual-layered 3D printed structure; dimensions of the layers: the dermal layer consists of 10 layers, while the epidermal layer has eight filaments, oriented perpendicularly with an interfilament spacing of 0.75 mm and a Z-axis increment of 0.08 mm per layer; microscopic view of the printed structure; dual-layered 10 × 10 mm 3D printed structure in culture; and the mechanically printed structure is noted for its optimal handling properties, which facilitate thorough characterization and assessment (adapted with permission from [247], Copyright 2019 Elsevier); (**C**): Assessment of the printability of acellular dermal matrix and GelMA hydrogels by fabricating identical structural patterns using these materials (adapted with permission from [248], Copyright 2021 Elsevier); (**D**): Macrographs and scanning electron microscopy (SEM) images of PCL-AMX, SG-rhEGF, and PCL-AMX-SG-rhEGF structures (adapted with permission from [249], Copyright 2024 Elsevier).

3D printing/bioprinting has arisen as an innovative technological approach in skin tissue engineering, creating structures by depositing cell-embedded bioinks layer by layer [244,245]. This approach includes several techniques, including DLP, extrusion bioprinting, inkjet printing and electrospinning. The materials commonly used in this field are natural polymers (e.g., collagen, alginate, HA, gelatin and fibroin) and synthetic polymers (e.g., PCL, PLGA, polyglycolic acid, polyurethanes, polycarbonates and PEGDA). These materials, often referred to as biopolymers, are biocompatible and biodegradable. In applications related to wound healing, bioinks can be integrated with antibiotic agents or antimicrobial peptides, as well as growth factors such as epidermal growth factor (EGF), fibroblast growth factor (FGF) or vascular endothelial growth factor (VEGF), to enhance cell stimulation, growth, proliferation and migration throughout the healing process [244,246]. The latest developments in wound care and skin regeneration involve embedding various cell types, including fibroblasts, hUVECs, keratinocytes and human umbilical cord mesenchymal stem cells (hUCMSCs), directly into bioinks. Liu et al. fabricated vascularized full-thickness skin substitute by printing an alginate-gelatin hydrogel to simulate the epidermis, and a phosphosilicate calcium bioglass (PSC)-alginate-GelMA hydrogel containing hUVECs and hUCMSCs to replicate the dermis (Figure 8A). For the bioprinting process, the printing speed was 10 mm/s with a filament orientation of 60°. They showed a marked enhancement in blood vessel formation and collagen deposition, demonstrating the effectiveness of these skin substitutes in reconstructing full-thickness skin injuries in rat models [241]. Also, with a view to developing a full-thickness skin substitute, Admane et al. employed extrusion-based 3D bioprinting to create a silk fibroin-gelatin construct containing fibroblasts to mimic the dermis, and a silk fibroin-gelatin layer with keratinocytes to replicate the epidermis (Figure 8B). The 3D bioprinted full-thickness skin model showed extensive keratinocyte migration and differentiation, mimicking reepithelialization. Analysis revealed similarities to native human skin, involving pathways related to skin development, extracellular matrix organization and keratinization [247]. Also, Jin et al. developed an advanced 3D bioprinted structure designed to mimic natural full-thickness skin, incorporating the epidermis, dermis and a vascular network. For the 3D bioprinting of the full-thickness functional skin model, the printing speed was set at 5 mm/s, with the air compressor pressure maintained at 0.2 MPa. This model utilised GelMA with HaCaTs for the epidermal layer, an acellular dermal matrix (ADM) with fibroblasts for the dermis and a GelMA mesh with hUVECs for the vascular network (Figure 8C). They demonstrated that this functional skin model not only enhanced cell viability and proliferation but also supported epidermal reconstruction in vitro. In vivo, the functional skin model maintained cell viability for at least one week and promoted wound healing, re-epithelization, dermal ECM secretion and angiogenesis, thereby improving wound healing quality [248].

Song et al. developed a bilayer skin scaffold incorporating drug delivery for the repair of full-thickness skin defects. The scaffold features an outer layer of amoxicillin (AMX)-loaded PCL nanofibers fabricated through electrospinning which functions as an antibacterial membrane mimicking the epidermis (Figure 8D). The inner layer, designed to replicate the dermis, is a hydrogel composed of sodium alginate and gelatin, infused with recombinant human epidermal growth factor (rhEGF) to maintain wound moisture and promote healing. The successful incorporation of AMX and rhEGF into the scaffold was demonstrated, with the scaffold exhibiting excellent physicochemical properties, effective drug release, and antibacterial activity. Both in vitro and in vivo evaluations revealed enhanced cell adhesion, proliferation and accelerated skin wound healing, alongside favourable biocompatibility. These results suggest that the scaffold holds considerable potential for skin regeneration applications [249].

Numerous studies have utilised 3D printing and bioprinting techniques, both with and without the integration of cellular therapies, to advance skin tissue engineering. A comprehensive overview of these studies is provided in Table 6.

### 5.5. Neural Tissue Engineering

The nervous system represents one of the most intricate and complex biological systems formed during development [261]. The human nervous system is divided into two primary components: the central nervous system, consisting of the brain and spinal cord, and the peripheral nervous system, which includes cranial and spinal nerves along with associated ganglia [262]. Traumatic injuries, including traumatic brain injury and spinal cord injury, as well as neurodegenerative diseases such as Alzheimer’s, Parkinson’s, Huntington’s and multiple sclerosis, pose major public health challenges, with limited treatment options available that mainly offer symptomatic relief. Autologous nerve graft transplantation is widely regarded as the optimal approach for addressing severe nerve injuries. Nonetheless, its clinical utility is constrained by several substantial issues, such as the restricted availability of suitable donor nerves and the risk of incompatibilities between the donor and recipient nerves [263,264,265]. Despite ongoing clinical advancements, fully effective therapies for neural regeneration are still in early stages, driving interest in neural tissue engineering [266]. Neural tissue engineering focuses on developing biological substitutes that integrate biomimetic 3D scaffolds with cells to improve neural tissue functionality. Advances in 3D printing technology have significantly impacted neural autograft engineering, enabling precise fabrication of tissue-engineered neural implants. Various 3D printing methods, such as extrusion-based printing, laser-assisted bioprinting, SLA and 4D printing, are increasingly employed to create accurately structured implants for nerve injury repair and to develop models and devices for in vitro neural tissue engineering.

The materials chosen for the printing of neural structures encompass biocompatible polymers (e.g., PCL and PU), composites (e.g., reduced graphene oxide (rGO)), and hydrogels (e.g., GelMa, HA, collagen and fibrin). These materials must meet specific requirements for printability and biocompatibility, as well as possess suitable physicochemical properties and mechanical strength [265].

**Figure 9:**
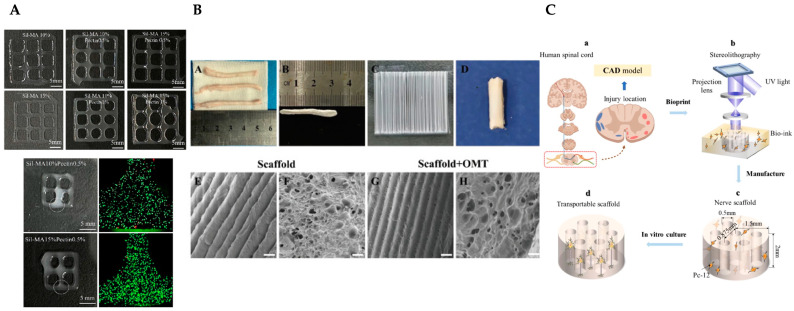
Advances 3D printing/bioprinting and cell therapies in neural tissue engineering: (**A**): Evaluation of bioink printability was conducted for various compositions of methacrylated silk fibroin and methacrylated silk fibroin/pectin hydrogels. Optical microscopy images were captured to examine the morphology, while a live/dead assay was performed on L929 cells embedded within 3D bioprinted constructs using bioinks composed of 10% methacrylated silk fibroin/0.5% pectin and 15% methacrylated silk fibroin/0.5% pectin (adapted with permission from [267], Copyright 2024 Elsevier); (**B**): Characterization of the scaffolds was conducted as follows: (B-A) Normal spinal cord tissue; (B-B) Decellularized spinal cord scaffold; (B-C) Polycaprolactone (PCL) microfiber structure; (B-D) Overall morphology of the 3D-bioprinted composite scaffold; (E–H) Microstructure of scaffolds visualized through scanning electron microscopy (SEM). The SEM images of both the scaffold alone and the scaffold combined with OMT reveal parallel microfibers of consistent thickness, with hydrogels adhering to the fibres. The interior of the scaffold exhibited uniform and densely packed pores, which are conducive to neural stem cell (NSC) growth and efficient nutrient exchange (adapted from [268]); (**C**): Schematic representation of the experimental procedure for spinal cord injury repair utilizing stereolithography apparatus (SLA) to create neural scaffolds. (a) Computer-aided design (CAD) to develop the scaffold model corresponding to the injured region for bioprinting; (b) Construction of neural scaffolds employing the SLA technique; (c) Utilization of the fabricated scaffolds for in vitro cell culture; (d) Assessment of the functionality of scaffolds following a period of in vitro cultivation (adapted with permission from [269], Copyright 2022 Elsevier).

Researchers have combined 3D printing/bioprinting with cellular therapies for neural tissue engineering. Lee et al. created photocrosslinkable methacrylated silk fibroin-pectin bioinks (Figure 9A). The structures were printed with an extrusion pressure ranging from 50 to 100 mm/min and a printing speed between 1 and 30 mm/s, and they exhibited tunable mechanical properties, favourable biocompatibility and an environment highly supportive of neural induction in 3D bioprinted constructs containing neural stem/progenitor cell spheroids [267].

For spinal cord injury treatment, Song et al. engineered scaffolds composed of PCL microfiber-reinforced spinal cord ECM hydrogels incorporating oxymatrine (OMT) using electrospinning techniques (Figure 9B). These scaffolds promoted neuronal differentiation of neural stem cells (NSCs) and suppressed astrocyte proliferation in vitro. In vivo, they promoted the recruitment of NSCs, stimulated neuronal growth, diminished glial scar formation and enhanced motor function recovery in rats with spinal cord injuries [268].

In turn, Lin et al. developed a model designed to forecast cell growth and distribution, aimed at reducing the need for empirical adjustments. They established a multiphysics model that integrates oxygen diffusion and substrate consumption dynamics within a rat adrenal medullary pheochromocytoma (pc-12) cell-laden nerve scaffold (Figure 9C). This model was used to simulate and forecast oxygen levels and cellular growth patterns. The scaffold was produced using SLA, and the distribution of cells was assessed through fluorescence staining to confirm the model. The findings demonstrated that the model effectively forecast cellular growth patterns [269].

Numerous studies have utilised 3D printing and bioprinting technologies, both with and without the integration of cellular therapies, to develop the area of neural tissue engineering. Table 7 provides a comprehensive overview of these investigations.

## 6. Limitations and Challenges

Despite the benefits of 3D printing/bioprinting and cellular therapies for regenerative medicine, there are several challenges that need to be addressed. Despite extensive research efforts in recent years, the clinical application of these technologies has been constrained. The lack of sufficient animal studies and the absence of viable 3D models in clinical trials underscore the need for further focus and development in these critical areas [280,281,282].

One of the current challenges is the advancement of functional vascular networks within bioprinted tissues and organs. Specifically, creating a vascular system that can seamlessly connect with the host’s native blood vessels is complicated by the complex structural architecture and the variability of tissue components [283,284,285]. Vascularization is crucial for providing nutrients and oxygen, which are vital for maintaining cell viability and ensuring tissue functionality. A robust, multi-level vascular network is necessary to support the long-term survival and growth of bioprinted organs, incorporating smooth muscle cells and hUVECs into the blood vessels [286]. To overcome challenges related to nutrient and oxygen transport, researchers have utilised proangiogenic factors, such as vascular endothelial growth factor (VEGF) and basic fibroblast growth factor (BFGF), to promote the development of microvessels. Additionally, incorporating endothelial cells into the culture medium has been shown to promote microvessel formation and the development of angiogenic sprouts in engineered constructs [287]. However, endothelial cells and angiogenic factors generally do not produce perfusable constructs in a fast way [288]. Bioreactors offer a solution by continuously supplying media to porous constructs, reducing the reliance on arterial scaffolds and large tissue samples. Despite this, these constructs often lack microvessels and are stored outside of bioreactors, which can compromise cell survival. Microfluidic systems represent a potential alternative for vascular network fabrication, though scaling these systems to larger physiological sizes remains a significant challenge [283].

The selection and sourcing of cells is another challenge in regenerative medicine. Cells used in bioinks must possess some key characteristics: high proliferative capacity, printability, functionality, safety and economic viability [286]. The choice of cells encapsulated in bioinks critically influences their differentiation potential and ability to develop into various lineages. Although live cells, such as stem cells, are very promising, their practical application is limited by issues related to availability and ethical concerns. Researchers have reported the successful integration of stem cells with bioprinting technologies [280]. An additional challenge is the mass production of cells, which needs substantial quantities of cells and increases the demand on in vitro expansion cultures. Addressing the cost-effectiveness of large-scale cell production is a crucial challenge [286]. Extended processing times and mechanical forces experienced during 3D printing can adversely affect cell viability by altering cell geometry and disrupting signalling pathways [289]. To address these issues, it is essential to enhance existing bioprinting techniques to minimise processing duration and to develop specialized buffers that can protect cells throughout the printing process.

In 3D bioprinting, choosing the most appropriate biomaterials is critical for the effective fabrication of tissues with clinical relevance [289,290]. Biomaterials are essential for providing structural support, maintaining cellular viability and ensuring long-term tissue integration. While numerous polymers traditionally utilised in 3D printing and tissue engineering have been explored for bioprinting due to their availability and previous applications, they may not always provide the optimal biological compatibility required for successful bioprinting outcomes [291]. These materials might display excessive biological reactivity, which can result in undesirable cellular interactions and premature or inappropriate differentiation of stem cells. For a bioink to be suitable for clinical applications, it must have specific characteristics, such as structural stability, the ability to support cell proliferation, and a degradation rate that matches the needs of tissue regeneration. Additionally, bioinks must be compatible with bioprinting technologies to facilitate rapid prototyping [13]. A major challenge is ensuring that printed structures are biocompatible and provide an appropriate environment for cell growth. Current research is focused on developing novel biopolymers and hydrogels that more accurately replicate the nanoscale features and responsive characteristics of the ECM and native tissue microenvironment [292]. However, these advanced materials often encounter compatibility issues with traditional bioprinting techniques. Many of these materials may lack the necessary structural integrity, leading to collapse if they are too soft [293]. One potential solution is to combine various materials to harness their individual strengths, such as merging the mechanical properties of more rigid materials with the cell-supportive and biocompatible features of softer ones [134,294].

Furthermore, the development and implementation of 3D printing and cell therapies in regenerative medicine pose significant financial challenges, which could prevent the widespread adoption of these technologies. The costs associated with research, development and clinical trials are substantial, and securing funding for these endeavours can be difficult. Moreover, the cost-efficiency of these technologies needs careful consideration, particularly in relation to the high expenses of 3D printers, cellular materials and associated computer software [295]. Generally, the costs of maintaining and scaling bioprinting technologies limit the rapid integration of 3D printing capabilities into clinical settings [292]. Another challenge is the size of bioprinted tissues. Currently, bioprinted constructs are typically small and consist of a limited number of cell types, which restricts their functionality and scalability [292,295,296,297]. Moreover, 3D printers are often constrained by their build volume, which limits the maximum size of bioprinted tissues and complicates the creation of entire 3D-printed organs [292].

Ethical considerations are increasingly influencing the development of 3D bioprinting technologies. The transition of 3D printing/bioprinting research from the laboratory to clinical applications raises both established ethical concerns, as discussed in the bioethics literature, and emerging issues specific to the unique features of these new treatments [298,299,300]. Several ethical concerns arise with the advancement of 3D printing/bioprinting technologies, including issues related to the sourcing of cells and tissues, as previously mentioned. Furthermore, the development of these technologies requires careful ethical consideration, particularly in research involving animal welfare and the design of human clinical trials. It is crucial to find a balance between the necessity of animal testing and the ethical treatment of research subjects, while thoroughly assessing the risks and benefits for human trials [301]. As bioprinting technologies evolve, ensuring equitable access becomes an ethical obligation. Addressing potential healthcare disparities and balancing the advantages of personalized medicine with fair healthcare delivery are significant challenges that must be addressed [302]. The rapid rate of the development of bioprinting has outpaced existing regulatory frameworks, resulting in gaps that need to be closed. Despite their complexity and time demands, valuable guidelines for these processes can be obtained from regulatory agencies such as the Food and Drug Administration (FDA) in the United States and the European Medicines Agency (EMA) in the European Union, especially regarding 3D-printed medical devices. For tissue bioprinting to achieve clinical translation, it is essential to establish a clear and defined regulatory pathway [303]. Additionally, challenges and concerns related to biosafety and liability arise when fabricating internal tissues and organs. The clinical translation of bioprinting techniques will depend on regulatory bodies’ thorough the evaluation of safety, efficacy and risk. Globally, regulatory authorities face challenges in addressing the potential and uncertain risks associated with 3D bioprinting, such as immune responses to bioinks or materials [292]. In the absence of specific regulations, the FDA is currently relying on the guidelines of the Center for Biologics Evaluation and Research (CBER) for 3D bioprinting products. These products require FDA approval, and adherence to regulatory guidelines is mandatory from the initial stages of product development. As the field advances, more 3D bioprinting products are likely to emerge, highlighting the need for more specific regulatory guidelines. Currently, only South Korea’s Ministry of Food and Drug Safety (MFDS) and Japan’s Pharmaceuticals and Medical Devices Agency (PMDA) have developed specific guidelines for 3D bioprinting. Thus, the global development of comprehensive regulations for 3D bioprinting techniques, bioinks and printers is of increasing importance [13].

These limitations and challenges highlight the complexity of integrating 3D printing with cellular therapies in regenerative medicine, emphasizing the need for continuous research and development in this field. Overcoming these obstacles requires transdisciplinary collaboration among cell biologists, engineers, physiologists and pharmaceutical industry partners to advance and expand the potential of this technology [304]. Despite the existing challenges, advancements persist, and it is anticipated that these technologies will increasingly impact the treatment of various diseases and conditions in the future. Ongoing research is progressively making the goal of creating safe and fully functional bioprinted tissues more achievable [286].

## 7. Current State and Future Outlook

The current state of clinical trials combining 3D printing/bioprinting and cellular therapies is promising, though they are still in their early stages. Numerous research groups are actively developing and testing new treatments utilizing these technologies, and several clinical trials have been initiated in recent years to assess their safety and efficacy.

Tissue engineering currently has broad applications, including the development of various tissues, cardiac, vascular, bone, skin, neural, cartilage and retinal tissues, among others. Conventionally, this method entails seeding cells onto a porous scaffold to promote their growth and, subsequently, promote tissue development [305]. The approach presents several advantages, including providing strong structural support with suitable degradation timing, regulating the cellular environment and allowing for effective nutrient and waste exchange between the scaffold and cells [306,307].

Bioprinting has opened up a whole new era in tissue regeneration, allowing the production of patient-specific autologous organs and tissues [18,308,309,310,311]. This rapid prototyping technique allows for the fabrication of intricate tissue and organ structures by precisely depositing living cells and biomaterials layer by layer, based on a CAD model. Using this approach, 3D constructs can be produced with high accuracy in terms of positioning and architecture, including shape, pore geometry and interconnectivity. This allows for the development of tissue and organ models that closely resemble the human body, with high reproducibility [312,313,314]. By simultaneously printing multiple cell types and biomaterials, bioprinting can replicate the structural and biochemical complexity of living tissues, creating a heterogeneous microenvironment at specified locations [128,315,316].

Although in vitro models have advanced significantly for developing new therapies, their application in surgical settings is still not fully realized. However, significant progress is being made in hydrogel design and the development of advanced technological tools. These advancements are bringing us closer to meeting the fidelity and safety standards required for bioprinted constructs to be used consistently and in a personalized way in patients in the near future [317].

Additionally, artificial intelligence (AI) is leveraging significant advances in AM for regenerative medicine, particularly in the production of personalized medical devices, implants and tissue engineering. AI algorithms optimize 3D printing parameters such as speed, temperature and layer thickness, increasing the precision and quality of printed structures [318].This optimization is crucial for the creation of patient-specific implants and prostheses, as well as for the development of 3D-printed tissues and organs, advancing the field of regenerative medicine [319]. In addition, AI-assisted 3D printing enables the manufacture of detailed models for surgical planning and testing, improving surgical results and precision [320]. Moving forward, researchers predict that AI will continue to revolutionize AM, enabling a more seamless use of the technology for complex designs in artificial organs, flexible electronics and wearable biosensors [321]. The synergy between AI and AM promises to unlock new possibilities for design, production and innovation in regenerative medicine [322].

The future of tissue biomaterials is expected to replicate not only the structural design and characteristics of organs and tissues but also their dynamic, functional behaviours [159,160]. The concept of time as the fourth dimension (4D) has gained prominence, in the context of bioprinting, introducing two key aspects: materials capable of deformation and structures that mature after printing [323,324]. This new approach to bioprinting addresses the complexity of the system, which is very important for fully understanding the behaviour of functional living materials during the post-processing stage [317].

## 8. Conclusions

The combination of 3D printing/bioprinting technologies with cellular therapies represents a significant advancement in regenerative medicine. Advances in this area are leading to the development of new functional tissues that closely resemble native tissues. This review has detailed the fundamental principles and applications of each technology individually and has elucidated how their convergence is pushing the boundaries of tissue engineering

This study underlines that the latest developments have markedly enhanced the capacity to produce complex 3D cellular structures presenting high precision. Innovations in bioprinting techniques, coupled with improvements in biomaterials and cellular engineering, have enabled more sophisticated control over tissue architecture and cellular organization. These advancements facilitate the creation of more accurate and functional tissue models, which hold promise for personalized regenerative therapies.

Nevertheless, there are still several critical barriers that need to be addressed, such as the development of functional vascular networks, regulatory and ethical issues and the advancement of suitable biomaterials.

The potential impact of these integrated technologies in regenerative medicine is expected to be high in the future. As the field progresses, solving these issues will be essential to realizing the full potential of 3D printing/bioprinting and cell therapies. Ongoing research and innovation in these areas is therefore expected to produce transformative advances in personalized medicine and tissue regeneration, ultimately improving outcomes for patients and advancing therapeutic options.

## Figures and Tables

**Table 1:** Advantages and drawbacks of each AM technique [22,23,24,33,42,43,44].

Printing Technology	Vat Photopolymerization	Material Extrusion	Power Bed Fusion	Material Jetting	Binder jetting	Directed Energy Deposition	Sheet Lamination
**Advantages**	High precision and good surface finish Capability to use transparent materials Relatively fast processing time Typically large build volumes	Widely adopted and cost-effective Exhibits favorable material properties Minimal material waste Relatively high fabrication speed User-friendly nature Capability to produce complex components	Relatively cost-effective Capable of integration into small-scale applications Broad compatibility with a wide range of materials Extensive material versatility	High precision High processing speed Minimal material waste Capability to produce multi-material and multi-color components within a single process	Diverse color options Wide range of material choices Rapid processing time Capability to process two materials simultaneously	High-quality components Speed is frequently compromised to achieve higher accuracy	High processing speed Cost-efficient fabrication Simplified material handling
**Drawbacks**	Relatively high cost Extended post-processing time Limited range of compatible materials Necessity for support structures during fabrication	Limited by nozzle radius Low precision and fabrication speed Requires consistent material pressure during operation Prone to delamination issues	Relatively low fabrication speed Materials often lack robust structural properties Size constraints limit the scalability of produced componentes High energy consumption during processing Surface finish quality is influenced by powder grain size Thermal stress and material degradation are common challenges	Support structures are frequently necessary Limited material options Frequent nozzle blockages Low viscosity and mechanical strength	Not always suitable for structural components due to the use of binder materials Extended post-processing time required High porosity and low surface quality	Post-processing may be required to achieve the desired effect Limited material availability Thermal stresses and the need for controlled atmospheric conditions	Low precision Prone to shrinkage during processing Generates a significant amount of material waste Delamination is a frequent issue

**Table 2:** Comparison of bioprinting techniques [60,72,73,74,75].

Printing Technology	Laser-Based Printing		Extrusion Printing	Inkjet Printing
	Stereolithography 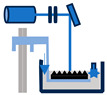	Selective Laser Sintering 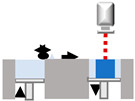	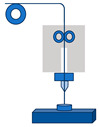	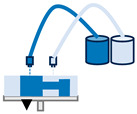
	High accuracy/resolution Printing time independent of complex geometries Fast fabrication speed Good vertical printability Smooth surface finish	No need for support structures Suitable for a wide range of materials High mechanical strength of printed constructs High productivity Ability to print complex structures	Relatively simple and cost-effective setup Vesrsatile material choices Ability to print with high cell densities and high viscosity biomaterials No need for support materials	Low cost and scalable High printable resolution/precision Fast fabrication speed Wide availability Ability to print low viscosity biomaterials Low material wastage Capable of depositing multiple cell types simultaneously
**Drawbacks**	Limited to photocurable biomaterials Potential cytotoxicity of photoinitiators Lack of printing multi-cells Need for support materials Expensive equipment and materials	Rough surface High equipment cost High energy consumption Difficulty in printing cell-laden constructs Slow fabrication speed Post-processing required to remove excess powder	Slow fabrication speed Low printable resolution/precision Possible nozzle clogging Potential shear stress-induced cell damage during extrusion	Lower mechanical strength of printed constructs Limited printable materials/requires low viscosity materials and low cell densities Need for support materials Potential clogging of print nozzles

**Table 3:** Recent advances in 3D printing/bioprinting and cellular therapies for cardiovascular tissue engineering. Abbreviations: human microvascular endothelial cells (hMVECs); mouse embryonic fibroblasts (mEFs); human cardiac-derived cardiomyocyte progenitor cells (hCMPCs); human umbilical vein endothelial cells (hUVECs); cardiomyocytes (H9c2 cells); induced pluripotent stem cell-derived cardiomyocytes (iCMs); human cardiac fibroblasts (CFs); human coronary artery endothelial cells (hCAECs); gelatin methacrylate (GelMA); decellularized cardiac extracellular matrix hydrogel (cECM); human cardiac progenitor cells (hCPCs); heart tissue-derived extracellular matrix (hdECM); neonatal rat cardiomyocytes (NRCMs); poly(ethylene glycol)-fibrinogen (PF); induced pluripotent stem cell-derived cardiomyocytes (iPSC-CMs); digital light processing (DLP); poly (glycerol sebacate) (PGS); poly (ℇ-caprolactone) (PCL); fused deposition modeling (FDM); not applicable (n/a); stereolithographic assembly (SLA); poly(ethylene glycol) dimethacrylate (PEGDMA); bone marrow-derived mesenchymal stem cells (BMSCs); mesenchymal stem/stromal cell (MSC); hyaluronic acid (HA); human embryonic stem cells (hESCs); early vascular cells (EVCs); hyaluronic acid glycidyl methacrylate (HAGM); neonatal rat cardiomyocytes (NRCMs).

3D Printing Technology	Biomaterials	Cellular Therapies	Application	Outcomes	Reference
Inkjet Printing	Fibrin gel	hMVECs	Microvasculature construction	The construction promoted hMVEC proliferation and microvasculature formation.	[177]
Extrusion 3D Bioprinting	NovoGel	mEFs	Aortic tissue construct	Support structure and mEF were successfully printed with the self-supporting approach.	[178]
	Alginate	hCMPCs	Construction with cardiogenic potential for use in vitro and in vivo	The printed hCMPCs were able to migrate in the alginate matrix while maintaining their functional properties.	[179]
	Alginate/Gelatin	hUVECs, H9c2 cells	Cardiac tissue engineering	A new angular structure that replicates the alignment of cardiac fibers demonstrates excellent cell viability for both hUVECs and H9c2 cells, substantial mechanical strength, and appropriate characteristics for dilation and degradation.	[180]
	Alginate/Gelatin	iCMs, CFs, hCAECs	Cardiac tissue patches	Epicardial transplantation using 3D bioprinted patches based on AlgGel resulted in improved cardiac function.	[181]
	GelMA, Alginate	hUVECs	Endothelialized myocardial tissues	New method for generating endothelialised fabricated organoids.	[182]
	GelMA	cECM, hCPCs	Cardiac tissue patches	Cardiac patches printed with cECM and GelMA demonstrated significantly higher viability of hCPCs and exhibited a 30-fold increase in the gene expression of cardiogenic genes related to patches made. exclusively of GelMA. In vivo studies on rats revealed vascularization over a period of 14 days.	[174]
	Collagen	hdECM, NRCMs	Cardiac tissue model	It promoted early differentiation and improved the maturation of cardiomyocytes in hdECM.	[183]
3D bioprinting with microfluidic printing head	Alginate, PF	iPSC-CMs, hUVECs	Vascularized cardiac tissue	The 3D cardiac tissue composed of iPSC-CMs presents a high orientation index due to the distinct geometries and vascular-like formations generated by hUVECs.	[173]
DLP-based 3D printing	PGS/PCL/Gelatin	hUVECs	Heart valve substitute	A crosslinked 3D valve with elastomeric characteristics.	[184]
FDM 3D printing	PGS/PCL	n/a	Myocardial remodeling	It improved and preserved heart function.	[185]
SLA	PEGDMA	BMSCs	Gel patch to damaged cardiac tissue	Application of the MSC-laden microchanneled gel patch resulted in enhanced ejection fraction, fractional shortening, and stroke volume.	[175]
Pneumatic-extrusion	Fibrinogen, gelatin, aprotinin, HA	hESCs-derived EVCs	Cardiac tissues	Spheroids derived from hESC-derived endothelial and EVCs offer greater potential for engineering complex vascular structures compared to single cells.	[186]
Micro-continuous optical printing	GelMA, HAGM	hiPSC-CMs	Cardiac micro-tissue (for drug testing)	The micro-tissue exhibited a well-organized sarcomere structure and a marked upregulation in the expression of maturity markers.	[187]
Pneumatic 3D printing	Fibrinogen, gelatin, aprotinin, glycerol, HA	NRCMs	Functional and contractile cardiac tissue constructs	They fabricated a structured construct exhibiting physiological and biomechanical properties comparable to those of native cardiac tissue.	[188]

**Table 4:** Recent developments in 3D printing/bioprinting and cellular therapies for bone tissue engineering. Abbreviations: selective laser melting (SLM); bone marrow-derived mesenchymal stem cells (BMSCs); beta-tricalcium phosphate (β-TCP); gelatin methacrylate (GelMA); Xonotlite (Sr-CSH); poly(lactic-co-glycolic acid) (PLGA); bone morphogenetic protein 2 (BMP-2); human umbilical cord mesenchymal stem cells (hUCMSCs); alkaline phosphatase (ALP); nano-hydroxyapatite (nHA); polycaprolactone (PLC); polylactic acid (PLA); cone beam computed tomography (CBCT); dental pulp stem cells (DPSCs); hydroxyapatite (HAp); Magnesium (Mg^2+^); Strontium (Sr^2+^); Zinc (Zn^2+^); not applicable (n/a); amorphous calcium phosphate (ACP); diacrylate poly(ethylene glycol) (PEGDA); digital light processing (DLP); tantalum (Ta); rat bone marrow mesenchymal stem cells (r-BMSCs); diamond (DO); rhombic dodecahedron (RD); iron (Fe); manganese (Mn); calcium (Ca); magnesium (Mg); titanium (Ti); niobium (Nb); zirconium (Zr); stromal cells from apical papilla (SCAPs); human umbilical vein endothelial cells (hUVECs).

3D Printing Technology	Biomaterials	Cellular Therapies	Application	Outcomes	Reference
SLM	Ti6Al4V, Matrigel	BMSCs	Mandibular bone defect reconstruction	Scaffold-Matrigel-BMSCs with enhanced bioactivity and mechanical properties for bone repair.	[198]
3D bioprinting	Alginate, β-TCP	MG-63 fibroblasts	Bone defects	10% alginate/β-TCP improved cells proliferation and alkaline phosphatase activity	[199]
	GelMA, Sr-CSH	BMSCs	Critical-size bone defects	GelMA-Sr–CSH scaffolds indicated complete regeneration of critical-size bone defects.	[201]
	Alginate, PLGA	BMP-2, hUCMSCs	Bone tissue engineering	hUCMSCs printing with Alginate/BMP-2 loaded into PLGA improved osteogenesis of the printed cells as demonstrated by higher rates of ALP activity, calcium deposition, expression of genes associated with osteogenesis, and mineralization when compared with controls.	[202]
	Alginate, collagen I, nHA	hBMSCs	In vitro model for investigating osteogenesis and the initial stages of tissue maturation	The integration of nHA and Col into the alginate allowed the formation of a bioink with ideal physical and biological characteristics, providing a promising 3D bioprinting platform for replicating bone tissue.	[203]
3D bioprinting and Fused Deposition Modeling	PCL and PLA		Periodontal defects	Using CBCT data from patients and employing fused deposition modelling, the printing process was straightforward and effectively produced personalized 3D models of periodontal defects.	[204]
3D bioprinting and extrusion	PCL, nHA, alginate, collagen I	DPSCs	Treatment of critical-size bone defects, targeted drug administration, and 3D models for bone tissue engineering	The alginate-nHA-Col hybrid scaffold demonstrated superior cell attachment, proliferation, and osteogenic differentiation in vitro compared to controls, while in vivo studies in a rat model confirmed its biocompatibility with minimal inflammatory responses.	[205]
Vat photopolymerization	HAp multi-substituted with Mg^2+^, Sr^2+^, and Zn^2+^ ions	n/a	Cancellous bone defects	It is a suitable technique for manufacturing highly complex trabecular structures for bone regeneration.	[200]
Extrusion 3D Bioprinting	PCL, ACP	n/a	Bone defects	It presented suitable properties, such as compressive strength, pore size, rigidity and repeatability (related to the interconnectivity between the pores).	[54]
	GelMA	DPSCs	Bone regeneration	DPSCs in GelMA bioprinted presented better osteogenic differentiation potential	[206]
	PCL, HAp, PEGDA	DPSCs	Bone regeneration	PCL/HANp/PEGDA revealed hydrophilic properties, suitable mechanical performance and significantly higher cell viability than the other groups.	[195]
DLP-based bioprinting	GelMA, dextran emulsion	BMSCs	Bone regeneration	It promoted the proliferation, migration, dissemination and differentiation of encapsulated BMSCs.	[207]
Laser Powder Bed Fusion	Ti6Al4V, Ta	r-BMSCs	Orthopedic clinical applications	It works well with the mechanics, supports the adhesion, growth and development of r-BMSCs into osteoblasts.	[197]
	PCL, HAp	BMSCs	Human mandibular trabecular bone regeneration	The biodegradable structures (with DO and RD elementary unit cell geometry) demonstrated their suitability as supports for bone regeneration.	[208]
Binder-jetting	Fe-Mn-Ca, Mg	n/a	Cranio-maxillofacial bone defects	The Fe-Mn and Fe-Mn-1Ca structures confirmed higher degradation from the addition of Ca to the 3D printed structures and good cytocompatibility.	[209]
Laser Directed Energy Deposition	Commercially pure Ti	n/a	Dental restoration	Commercially pure grade 4 titanium produced by Laser Directed Energy Deposition has a higher mechanical response than other techniques, which can be attributed to the modification of the microstructure inherent in the process.	[210]
	Ti-Nb, Ti-Zr-Nb	n/a	Orthopedic and dental applications	Ti-35Zr-25Nb presented a lower modulus of elasticity, higher hardness, good corrosion resistance and in vitro biocompatibility.	[211]
Sheet lamination	AISI 302	n/a	Bone regeneration	The scaffold prototype was designed and fabricated with the parameters selected through experimental tests and using the mathematical model.	[212]
Laser-assisted bioprinting	BioRoot RCS^®^	SCAPs, hUVECs	Bone regeneration	The application of laser-assisted bioprinting techniques with this ink failed to provide complete bone repair, whether the SCAPs were printed in direct proximity or not.	[213]

**Table 5:** Recent advances in 3D printing/bioprinting and cellular therapies for liver tissue engineering. Abbreviations: digital light processing (DLP); methacrylated gelatin (GelMA); decellularized extracellular matrix (dECM); human-induced hepatocyte (hiHep cells); hyaluronic acid (HA); primary mouse hepatocytes (PMHs); human induced pluripotent stem cells (hiPSCs); human embryonic stem cells (hESCs); human pluripotent stem cells (hPSCs); induced pluripotent stem cells (iPSC); 3D bioprinted hepatorganoids (3DP-HOs); hepatocellular carcinoma (HCC); poly (ℇ-caprolactone) (PCL); human bone marrow-derived mesenchymal stem cells (BMMSCs); human hepatocellular carcinoma (hepG2); cellulose nanocrystals (CNCs); bioink with alginate (1%), cellulose nanocrystal (3%), and gelatin methacryloyl (5%) (135ACG); undifferentiated hepatocyte cell line (HUH7); primary rat hepatocytes cells (PrHCs); human umbilical vein endothelial cells (hUVECs); human lung fibroblasts (hLFs); human extracellular matrix (hECM); human hepatic stellate cell line (L × 2); primary fetal activated hepatic stellate cells (aHSC).

3D Printing Technology	Biomaterials	Cellular Therapies	Application	Outcomes	Reference
DLP bioprinting	GelMA	dECM, hiHep cells	Hepatic functional restoration	It was found that the addition of hepatic dECM to the biotints improved printing capacity and cell viability. Furthermore, hiHep cells exhibited increased spreading and enhanced functional performance within the liver microtissue.	[231]
3D bioprinting	HA, alginate, gelatin	dECM, PMHs	Functional in vitro liver tissue models	The inclusion of dECM enhanced the hepatic function of hepatocyte spheroids.	[232]
	Alginate	hiPSCs, hESCs	Mini-liver tissue structures	Using this approach, researchers have been successfully bioprinting hPSCs whilst preserving their pluripotency or directing their differentiation towards specific cell types.	[233]
	NovoGel 2.0	iPSC-derived hepatocyte	Functional in vitro liver tissue models	It demonstrated a method for rapidly fabricating multicellular 3D liver constructs in a multi-well format, displaying critical liver functions including albumin synthesis, cholesterol biosynthesis, production of fibrinogen and transferrin and inducible activities of CYP 1A2 and CYP 3A4.	[219]
	Alginate	HepaRG cells	Hepatorganoids: Liver tissue model	The implantation of 3DP-HOs markedly enhanced the survival rates of mice experiencing liver failure.	[226]
	Gelatin, Alginate	Primary HCC cell lines	In vitro models for patient-specific drug screening for HCC	The generated models preserved the key characteristics of the original HCCs, including consistent biomarker expression, stable genetic alterations and maintained expression profiles.	[227]
	PCL	dECM, BMMSCs, HepG2	3D cell printing-based liver tissue engineering	The liver dECM bioink proved to have excellent printing capacity without significant cell death during the process. It also improved stem cell differentiation and HepG2 cell function.	[234]
	Alginate	HepG2	Reconstruction of liver tissues or organs	The cells proliferated well in the scaffold and the expression of liver-specific genes increased.	[229]
	GelMA, alginate, CNC	NIH/3T3 fibroblasts, hepG2	Bicellular liver lobule-mimetic structures	NIH/3T3 cells proliferated effectively within the rigid 135ACG matrix and aligned along the boundary between 135ACG and GelMA, demonstrating durotaxis. In contrast, HepG2 cells formed spheroids exclusively within the softer GelMA matrix. The 3D co-culture of HepG2 and NIH/3T3 cells exhibited increased albumin secretion, indicating that the enhancement in liver cell functionality may be due to soluble factors in the environment.	[230]
Pneumatic extrusion	Gelatin	HUH7	Model hepatocyte system	It has been proved that the scaffold geometry, using well-defined gelatin constructs, modulates hepatocyte function.	[228]
	PCL, collagen	PrHCs, hUVECs, hLFs	Liver tissue engineering	The 3D cell printed construct comprising a capillary-like network enhanced the protein secretion and metabolism of PrHCs. It demonstrated a great potential for functional liver tissue regeneration.	[235]
	Alginate, gelatin	hECM, human HepaRG liver cells	3D liver model for infection and transduction studies	It was demonstrated that supplementing an alginate/gelatin bioink with hECM enhanced cell viability and hepatic metabolic function in a 3D-printed humanized liver model.	[236]
Extrusion bioprinting	Alginate	HepG2/C3A, EA.hy926	Hepatic lobules within a highly vascularized construct	The successful bioprinting of hepatic lobules with a complex, highly vascularized architecture resulted in enhanced albumin secretion, urea production and elevated levels of albumin, MRP2, and CD31 proteins. Additionally, cytochrome P450 enzyme activity was significantly greater in these constructs compared to other groups.	[237]
	HA, collagen I	L × 2, aHSC	3D bioprinted liver model	The formulations seemed to facilitate cell viability, in line with the biomatrices used. The bioinks incorporating primary human hepatocytes were evaluated over two weeks, demonstrating sustained production of urea and albumin while showing a responsive reaction to acetaminophen.	[238]
	Alginate, CNCs	Fibroblasts, Human hepatoma cells	Liver-mimetic structures	This bioink showed excellent shear-thinning property, extrudability and shape fidelity after the deposition. The bioprinting caused minimal cell damage.	[239]

**Table 6:** Recent advances in 3D printing/bioprinting and cellular therapies for skin tissue engineering. Abbreviations: strontium silicate (SS); matrix hydrogel with 2.8% of gellan gum, 1.6% of alginate, and 2.8% of methyl cellulose (GAM); human dermal fibroblasts (hDFs); human umbilical vascular endothelial cells (hUVECs); murine umbilical vein endothelial cells (MUVECs); dextran (Dex); vascular endothelial (VEGF); gelatin (Gel); keratin (Kr); not applicable (n/a); bi-layer (BL); poly (ℇ-caprolactone) (PCL); amoxicillin (AMX); external human epidermal growth factor (rhEGF); Digital light processing (DLP); gelatin methacrylate (GelMA), hyaluronic acid (HA); N-(2-aminoethyl)-4-(4-(hydroxymethyl)-2-methoxy-5-nitrosophenoxy) butanamide (NB); lithium phenyl-2,4,6-trimethylbenzoylphosphinate (LAP); human skin fibroblasts (hSFs); silk fibroin (SF); 4-arm polyethylene glycol acrylate (PEG4A); platelet-rich plasma (PRP); dermal fibroblasts (DFs); epidermal stem cells (ESCs); acellular dermal matrix (ADM); polyurethane (PU); endothelial progenitor cells (EPCs); human keratinocytes (hKCs); pectin methacrylate (PecMA); human neonatal dermal fibroblasts (hNDFs); extracellular matrix (ECM); human endothelial cells (hECs); human placental pericytes (hPCs); phosphosilicate calcium bioglasses (PSCs); human umbilical cord mesenchymal stem cells (hUCMSCs); poly-(lactic-co-glycolic acid) (PLGA).

3D Printing Technology	Biomaterials	Cellular Therapies	Application	Outcomes	Reference
3D bioprinting	Collagen, SS, GAM	hDFs, hU-VECs, MUVECs, BALB/3T3 fibroblast	Vascularized Skin substitute	Collagen-2SS-GAM demonstrated significant capabilities in promoting angiogenesis, integrating with host tissue and facilitating wound repair in vivo.	[250]
Extrusion-based 3D printer and electrospinning	Dex-VEGF, Gel-Kr	n/a	Construct to accelerate wound healing	The BL-VEGF construct is identified as an optimal candidate for enhancing the healing process of full-thickness skin wounds.	[251]
	PCL, AMX, alginate, gelatin	rhEGF	Repair defective skin tissue and wound healing	The PCL-AMX@SG-rhEGF scaffold demonstrated superior drug release capabilities and significant antibacterial properties. Both in vitro and in vivo analyses indicated that the scaffold facilitates cell adhesion and proliferation, accelerates skin wound healing and exhibits favourable biocompatibility.	[249]
DLP-based 3D printing	GelMA/HA, NB/LAP	hSFs, hUVECs	Functional living skin	The bioink exhibited rapid gelation, customizable mechanical properties, high biocompatibility and effective tissue adhesion. In vivo studies in large animal models showed that the bioink elicited an immediate defence response and was particularly effective in enhancing dermal regeneration, including the development of skin appendages.	[252]
	SF, PEG4A	NIH 3T3 fibroblas, keratinocytes	Artificial skin model	SF-polyethylene glycol hydrogels presented higher cell proliferation, and the thickest keratin layer was produced with SF-PEG4A hydrogels when compared to PEG4A alone.	[253]
Extrusion-based bioprinting (in situ)	Alginate, gelatin, PRP	DFs, ESCs	Wound repair	The addition of PRP enhanced the cellular behaviour of the cells, regulated vascular endothelial cell tube formation and macrophage polarisation in a paracrine manner. In in situ bioprinting, incorporating PRP accelerated high-quality wound closure, regulated inflammation and started angiogenesis more effectively than using alginate-gelatin bioink.	[254]
Extrusion-based bioprinting	GelMA	ADM, HaCaTs, hUVECs, fibroblasts	Functional skin model	The in vivo results showed that the functional skin model stimulated wound healing and re-epithelialisation, enhanced the production of dermal extracellular matrix, stimulated angiogenesis and overall improved the quality of wound repair.	[248]
	PU, gelatin	Fibroblasts, keratinocytes, EPCs	Skin tissue engineering.	Curvilinear-bioprinted hydrogel showed superior structural integrity over planar-bioprinted hydrogel. In the treatment of large and irregular rat skin wounds, the curvilinear-bioprinted tri-cell-laden hydrogel achieved complete repair within 28 days.	[255]
	Alginate, honey	3T3 fibroblast	Skin tissue engineering	The incorporation of honey resulted in bioprinted scaffolds with enhanced cell proliferation, without any visible decrease in printability.	[256]
	SF, gelatin	hDFs, hKCs	Full-thickness skin constructs	A silk and gelatine bioink has been printed that can be used to recapitulate a series of biological and design parameters comparable to human skin.	[247]
3D Bioprinting	PecMA	hNDFs	Biomimetic skin constructs	The hydrogels exhibited significant versatility, enabling precise adjustment of their rheological and viscoelastic properties. Additionally, they created an optimal microenvironment that supported the deposition of endogenous ECM by the entrapped hNDFs, which is rich in collagen and fibronectin.	[257]
	Rat tail type I collagen	hDFs, hKCs, hECs, hPCs,	Multilayered vascularized human skin grafts	In in vitro studies, hKCs grow and develop into a multi-layered barrier, while hECs and hPCs form interconnected microvascular networks. In vivo, the presence of hPCs in the printed dermal tissue increased the integration of the graft with the host’s microvessels and supports the generation of an epidermal network.	[258]
	PSCs, alginate, gelMA, gelatin	hUVECS, hUCMSCs	Skin tissue grafts	The incorporation of PSCs revealed enhanced cell proliferation and elevated expression of angiogenesis-related genes in vitro. In vivo experiments showed an improved wound healing effect, characterized by an increase in angiogenesis and collagen deposition.	[241]
Pneumatic-assisted extrusion freeforming	Alginate, gelatin	hSFs	Bioactive dermal substitute scaffold	The three-phase crosslinking technique can produce dermal substitute supports with physicochemical and biological properties suitable to be used in skin tissue engineering.	[259]
Inkjet printing	PLGA, prednisolone	n/a	Personalized dermal patches for treatment of skin diseases	The first steps have been made towards the concept of manufacturing personalized patches by inkjet printing that can be made for poorly soluble pharmaceuticals. The model drug prednisolone was successfully processed and printed in the format of a nanosuspension. The PLGA nanoparticles and the patches produced demonstrated a prolonged release of the pharmaceutical.	[260]

**Table 7:** Recent advances in 3D printing/bioprinting and cellular therapies for neural tissue engineering. Abbreviations: methacrylated silk fibroin (SilMA); pectin methacryloyl (PecMA); silk fibroin (SF); neural stem/progenitor cells (NSPCs); polyurethane (PU); neural stem cells (NSCs); hyaluronic acid (HA); neural progenitor cells (NPCs); poly(lactic-co-glycolic acid) (PLGA); primary human dermal fibroblasts (phDFs); vascular endothelial growth factor (VEGF); murine neural stem cells (C17.2); interferon-gamma (IFN-γ); poly (ℇ-caprolactone) (PCL); oxymatrine (OMT); polyethylene glycol diacrylate (PEGDA); digital light processing (DLP); gelatin methacrylate (GelMA); Poly(3,4-ethylenedioxythiophene) (PEDOT); not applicable (n/a); chondroitin sulphate methacrylate (CSMA); bone marrow-derived mesenchymal stem cells (BMSCs); stereolithography (SLA); rat adrenal medullary pheochromocytoma (pc-12); reduced graphene oxide (rGO).

3D Printing Technology	Biomaterials	Cellular Therapies	Application	Outcomes	Reference
Extrusion-based 3D Bioprinting	SilMA, Pectin, PecMA, SF	NSPCs	Neural tissue engineering applications or in vitro brain models	The SilMA/pectin biotints showed adjustable mechanical properties, biocompatibility and a highly propitious environment for neural induction.	[267]
	PU	NSCs	Neural tissue engineering	NSCs proliferated and differentiated favourably in PU2 bioprinted hydrogels. In the in vivo model of neural injury in the zebrafish embryo, the injection of hydrogels loaded with NSCs facilitated the repair of the damaged central nervous system. Additionally, the functionality of adult zebrafish with traumatic brain injury was recovered following the injection of constructs with NSCs.	[270]
	Gelatin, alginate, fibrinogen, nanofibrillate cellulose, matrigel, HA	NPCs, astrocytes	Study the connection between human neuronal networks, model pathological processes and provide a platform for drug testing.	Imprinted neuronal progenitors differentiate into neurons and form functional neuronal circuits within and among tissue layers with specificity within weeks.	[271]
Laser Assisted Bioprinting	PLGA	NE-4C	Artificial neural tissue constructs	The findings of the study showed that the topological clue presents a relevant role in the development of clusters on the support, but that the bioprinted laminin dots appeared to regulate the strength of the bond between them, paving the way for controlling the functional morphology of artificial neural tissue constructions.	[272]
3D Bioprinting	PU	FoxD3 plasmids, phDFs	Personalized drug screening or neuroregeneration.	The phDFs embedded with FoxD3 in the thermo-responsive PU hydrogel exhibited the capacity to undergo reprogramming and develop into a neural tissue-like structure within 14 days post-induction.	[273]
	Collagen, fibrin gel	VEGF, C17.2	Neural tissue regeneration	The bioprinting of fibrin gel containing VEGF facilitated the prolonged release of growth factors on the collagen scaffold.	[274]
3D printing technology based on low-temperature extrusion	Collagen, chitosan	IFN-γ, NSC-derived exosomes	Neurological recovery after traumatic brain injury	The 3D printed collagen/chitosan structure exhibited favourable biological and mechanical characteristics, creating an optimal microenvironment for NSC differentiation and exosome secretion. Additionally, this scaffold played a role in various pathological processes following traumatic brain injury in rats, significantly improving neurofunctional recovery.	[275]
Electrospinning	PCL, OMT, gelatin, PEGDA, β-cyclodextrin	Spinal cord extracellular matrix	Treatment of spinal cord injuries	The manufactured scaffolds led to the differentiation of NSCs into neurons while suppressing their conversion into astrocytes. They established an optimal microenvironment for spinal cord tissue regeneration in vivo and directed axonal growth. Additionally, the transplantation of these scaffolds, combined with OMT, improved motor function recovery in rats with spinal cord injuries.	[268]
DLP printing	GelMA, chitosan, PEDOT	n/a	Neural tissue repair	The GelMA/Chitosan-PEDOT hydrogel showed good and stable electrical conductivity, enhanced mechanical strength and noticeable biocompatibility. In vivo tests showed that the hydrogels promoted nerve regeneration and helped muscle recovery in the repair of sciatic nerve defects.	[276]
Microextrusion-based 3D bioprinting	GelMA, PEGDA, PEDOT, CSMA	NSCs	Nerve injury repair	3D bioprinted electroconductive hydrogel showed biocompatibility, suitable mechanical strength and good conductivity, thus promoting the adhesion, growth and proliferation of NSCs.	[277]
Additive-lathe 3D bioprinting	GelMA, PEGDA	BMSCs	Peripheral nerve injury repair	An integrated two-layer nerve conduit was developed, in which the BSMCs within the printed nerve conduits demonstrated excellent cell viability and extended cellular morphology. In vitro studies with PC12 cells revealed that the growth of neurons is significantly enhanced in nerve conduits embedded with BMSCs.	[278]
SLA	GelMA, PEGDA	Pc-12	Multi-physical model for cell-laden nerve scaffolds	This model enables the prediction of key regions for cell growth within neural scaffolds during in vitro culture experiments. By identifying these primary growth areas, the model allows for targeted increases in oxygen concentration in these regions, thereby enhancing cell density and distribution.	[269]
Electrohydrodynamic jet	PCL, rGO	PC-12	Peripheral nerve injury repair	The addition of rGO to scaffolds results in softer materials that enhance neural differentiation. In vitro studies with PC12 cells show increased cell proliferation and improved support for neural differentiation in PCL/rGO scaffolds compared to PCL-only scaffolds, as confirmed by RT-PCR and immunocytochemistry analyses.	[279]

## Data Availability

The data that support the findings of this study are available from the corresponding author on request.

## Abbreviations

2D	Two-dimensional
132ACG	Bioink with alginate (1%), cellulose nanocrystal (3%), and gelatin methacryloyl (5%)
2PP	Two-photon polymerization
3D	Three-dimensional
3DP-HOs	Three-dimensional bioprinted hepatorganoids
4D	Four-dimensional
ACP	Amorphous calcium phosphate
AD	Additive manufacturing
ADM	Acellular dermal matrix
aHSC	Primary foetal activated hepatic stellate cells
ALP	Alkaline phosphatase
AMX	Amoxicillin
ASTM	American Society for Testing and Materials
BFGF	Basic fibroblast growth factor
BJ	Binder jetting
BL	Bi-layer
BMP-2	Bone morphogenetic protein 2
BMSCs	bone marrow-derived mesenchymal stem/stromal cells
C17.2	Murine neural stem cells
Ca	Calcium
CAD	Computer-aided design
CBER	Centre for Biologics Evaluation and Research
cECM	decellularized cardiac extracellular
CFs	Human cardiac fibroblasts
CLIP	Continuous light interface production
CNC	Cellulose nanocrystal
CSMA	Chondroitin sulphate methacrylate
dECM	Decellularized extracellular matrix
DEP	Directed energy deposition
Dex	Dextran
DFs	Dermal fibroblasts
DIW	Direct ink writing
DLP	Digital light processing
DMLS	Direct metal laser sintering
DO	Diamond
DPSCs	Dental Pulp stem/stromal cells
EBM	Electron beam melting
ECM	Extracellular matrix
EMA	European medicines agency
EPCs	Endothelial progenitor cells
ESCs	Embryonic stem cells
ESCs	Epidermal stem cells
EVCs	Early vascular cells
FDA	Food and Drug Administration
FDM	Fused deposition modelling
Fe	Iron
FFF	Fused filament fabrication
GAM	Matrix hydrogel with 2.8% of gellan gum, 1.6% of alginate, and 2.8% of methyl cellulose
Gel	Gelatin
GelMA	Gelatin methacrylate
H9c2	Cardiomyocytes
HA	Hyaluronic acid
HAGM	Hyaluronic acid-gelatine methacrylate
HAp	Hydroxyapatite
Hap/β-TCP	Biphasic calcium phosphate system
hCAECs	Human coronary artery endothelial cells
HCC	Hepatocellular carcinoma
hCMPCs	Human cardiac-derived cardiomyocyte progenitor cells
hCPCs	Human cardiac progenitor cells
hdECM	Heart tissue-derived extracellular matrix
hDFs	Human dermal fibroblasts
hECM	Human extracellular matrix
hECs	Human endothelial cells
hepG2	Human hepatocellular carcinoma
hESCs	Human embryonic stem cells
hiHep	Human-induced hepatocyte
hiPSCs	Human induced pluripotent stem cells
hKCs	Human keratinocytes
hLFs	Human lung fibroblasts
hMVECs	Human microvascular endothelial cells
hnDFs	Human neonatal dermal fibroblasts
hPCs	Human placental pericytes
hPSCs	Human pluripotent stem cells
hSFs	Human skin fibroblasts
hUCMSCs	Human umbilical cord mesenchymal stem cells
HUH7	Undifferentiated hepatocyte cell line
hUVECs	Human umbilical vein/vascular endothelial cells
iCMs	Induced pluripotent stem cell-derived cardiomyocytes
IFN-γ	Interferon-gamma
iPSC-CMs	Induced pluripotent stem cell-derived cardiomyocytes
iPSCs	Induced pluripotent stem cells
ISO	International Standard Organization
Kr	Keratin
L x 2	Human hepatic stellate cell line
LAP	Lithium phenyl-2,4,6-trimethylbenzoylphosphinate
LOM	Laminated object manufacturing
mEFs	Mouse embryonic fibroblasts
MFDS	Ministry of Food and Drug Safety
Mg	Magnesium
MJ	Material jetting
Mn	Manganese
MSCs	Mesenchymal stem/stromal cells
MUVECs	Murine umbilical vein endothelial cells
n/a	Not applicable
Nb	Niobium
NB	N-(2-aminoethyl)-4-(4-(hydroxymethyl)-2-methoxy-5-nitrosophenoxy) butanamide
NPCs	Neural progenitor cells
NRCMs	Neonatal rat cardiomyocytes
NSCs	Neural stem cells
NSPCs	Neural stem/progenitor cells
OMT	Oxymatrine
PBF	Powder bed fusion
pc-12	Rat adrenal medullary pheochromocytoma
PCL	Poly (ℇ-caprolactone)
PecMA	Pectin methacrylate
PEDOT	Poly(3,4-ethylenedioxythiophene)
PEEK	Polyether ether ketone
PEG	Polyethylene glycol
PEG4A	4-arm polyethylene glycol acrylate
PEGDA	Diacrylate poly (ethylene glycol)
PEGDMA	Poly (ethylene glycol) dimethacrylate
PEGMA	Poly (ethylene glycol) methacrylate
PF	PEG-Fibrinogen
PF	Poly (ethylene glycol)-fibrinogen
PGA	Poly (glycolic acid)
PGS	Poly (glycerol sebacate)
phDFs	Primary human dermal fibroblasts
PLA	Poly(l-lactic) acid
PLGA	Poly (lactic-co-glycolic) acid
PMDA	Pharmaceuticals and Medical Devices Agency
PMHs	Primary mouse hepatocytes
PrHCs	Primary rat hepatocytes cells
PRP	Platelet-rich plasma
PSC	Phosphosilicate calcium bioglass
PSCs	Phosphosilicate calcium bioglasses
PU	Polyurethane
PVP	Polyvinylpyrrolidone
r-BMSCs	Rat bone marrow mesenchymal stem cells
RD	Rhombic dodecahedron
rGO	Reduced graphene oxide
rhEGF	External human epidermal growth factor
SCAPs	Stromal cells from apical papilla
SF	Silk fibroin
SilMA	Methacrylated silk fibroin
SLA	Stereolithography
SLM	Selective laser melting
SLS	Selective laser sintering
Sr-CSH	Xonotlite
SR^2+^	Strontium
SS	Strontium silicate
Ta	Tantalum
Ti	Titanium
Ti6AI4V	Titanium alloy
UAM	Ultrasonic additive manufacturing
UV	Ultraviolet
VEGF	Vascular endothelial
XG	Xanthan gum
Zn^2+^	Zinc
Zr	Zirconium
β-TCP	Beta-tricalcium phosphate
